# Computational model of precision grip in Parkinson's disease: a utility based approach

**DOI:** 10.3389/fncom.2013.00172

**Published:** 2013-12-02

**Authors:** Ankur Gupta, Pragathi P. Balasubramani, V. Srinivasa Chakravarthy

**Affiliations:** Computational Neuroscience Laboratory, Department of Biotechnology, Indian Institute of Technology MadrasChennai, India

**Keywords:** precision grip, Parkinson's disease, basal ganglia, reinforcement learning, decision making

## Abstract

We propose a computational model of Precision Grip (PG) performance in normal subjects and Parkinson's Disease (PD) patients. Prior studies on grip force generation in PD patients show an increase in grip force during ON medication and an increase in the variability of the grip force during OFF medication (Ingvarsson et al., 1997; Fellows et al., 1998). Changes in grip force generation in dopamine-deficient PD conditions strongly suggest contribution of the Basal Ganglia, a deep brain system having a crucial role in translating dopamine signals to decision making. The present approach is to treat the problem of modeling grip force generation as a problem of action selection, which is one of the key functions of the Basal Ganglia. The model consists of two components: (1) the sensory-motor loop component, and (2) the Basal Ganglia component. The sensory-motor loop component converts a reference position and a reference grip force, into lift force and grip force profiles, respectively. These two forces cooperate in grip-lifting a load. The sensory-motor loop component also includes a plant model that represents the interaction between two fingers involved in PG, and the object to be lifted. The Basal Ganglia component is modeled using Reinforcement Learning with the significant difference that the action selection is performed using utility distribution instead of using purely Value-based distribution, thereby incorporating risk-based decision making. The proposed model is able to account for the PG results from normal and PD patients accurately (Ingvarsson et al., 1997; Fellows et al., 1998). To our knowledge the model is the first model of PG in PD conditions.

## Introduction

Precision grip (PG) is the ability to grip objects between the fore-finger and thumb (Napier, 1956). Successful performance of PG requires a delicate control of two forces (grip force, *F*_*G*_, and lift force, *F*_*L*_) exerted by two fingers on the object. In a grip-lift task *F*_*G*_ is kept sufficiently high to couple *F*_*L*_ with the object via the agency of friction between the object and the fingers. An optimal *F*_*L*_ is also required to overcome the object's weight and lift it off the surface of the table on which it rests. These forces (*F*_*L*_ and *F*_*G*_) are thought to be generated in parallel by different subsystems in the brain (Ehrsson et al., 2001, 2003). The critical *F*_*G*_at which the object slips is called the slip force (*F*_slip_) and the difference between the actual steady state *F*_*G*_(*SGF*), used in a successful lift, and *F*_slip_ is known as the safety margin (*SM* = *SGF* − *F*_slip_). Johansson and Westling (1988) demonstrated the SM in controls to be 40–50% of slip force (Johansson and Westling, 1988). A high SM is employed to prevent the object from slipping due to internal (accelerations due to arm motion) (Werremeyer and Cole, 1997) and external (random changes in object load) perturbations (Eliasson et al., 1995)—motor activity is optimized for the internal perturbations and this optimality is lost on the addition of an external perturbation external perturbation (Charlesworth et al., 2011; Sober and Brainard, 2012; Wolpert and Landy, 2012). An excessive SM is undesirable as it would cause muscle fatigue and may even lead to crushing of the object.

SM in grip force is a crucial and defining parameter of PG performance. *F*_slip_ serves as the threshold below which the object cannot be lifted. Human subjects operate at *F*_*G*_ that is much higher than *F*_slip_; operation at a small SM makes the gripping unstable. Therefore, this need to operate sufficiently far from the border of instability may be regarded as a strategy for minimizing risk. The need for a large SM indicates that concepts from theories of risk-dependent decision making (DM) may be applied to understand PG performance (Bell, 1995; D'Acremont et al., 2009). By definition, risk is the variance in reward outcome (Bell, 1995; D'Acremont et al., 2009). In the context of PG performance, a reward may be thought to be associated with successful lifts. The risk is maximum close to *F*_slip_ and expected reward (value) saturates for grip forces much greater than *F*_slip_. Optimal PG consists of maximizing average reward while minimizing risk. The present study approaches the problem of PG in terms of risk minimization and describes it within the framework of Reinforcement Learning (RL). The model is used to explain PG performance in both controls and Parkinson's disease (PD) patients.

PG studies in PD patients show a remarkable difference in SM patterns between PD patients and controls (Ingvarsson et al., 1997; Fellows et al., 1998). PD patients were shown to be capable of storing and recalling previous lift parameters (Muller and Abbs, 1990; Ehrsson et al., 2003). This allows them to scale *F*_*G*_ when the load force on the object changes (Gordon et al., 1997; Fellows et al., 1998). Interestingly, even though *F*_*G*_ scaling is preserved, scientific community is divided on the question of sensory deficits in PD patients being a cause for their altered SM. A sensory deficit would lead to suboptimal sensory-motor coordination. Some studies support the above theory of sensory deficit in PD (Moore, 1987; Schneider et al., 1987; Klockgether et al., 1995; Jobst et al., 1997; Nolano et al., 2008) and others (Gordon et al., 1997; Ingvarsson et al., 1997) reject it. Ingvarsson et al. (1997) demonstrated that the controls and PD OFF patients generated nearly similar *SGF*s. A higher *SGF* was generated in PD ON (L-Dopa Medication) case when the lifted object was covered with silk, suggesting a higher safety margin in PD ON condition (Ingvarsson et al., 1997). It has been suggested that this increase in *SGF* may be due to L-Dopa induced hyperkinesias (Ingvarsson et al., 1997; Gordon and Reilmann, 1999). In another study, Fellows et al. (1998) observed that PD ON subjects show a higher *SGF* than controls, but there was no mention of PD OFF results (Fellows et al., 1998). Reports also suggest a considerably higher *SGF* variance in PD OFF condition when compared to controls and PD ON condition (Ingvarsson et al., 1997). This may indicate the importance of the concepts of *risk sensitivity* in understanding the *SGF* in controls, PD ON and PD OFF conditions. Furthermore, recent evidence suggests that risk-takers are less prone to Parkinson's disease (Sullivan et al., 2012). PD medications such as L-Dopa (Ehrsson et al., 2003) and dopamine agonists (Claassen et al., 2011) increase impulsivity and risk-seeking behavior in PD patients. PD subjects under medication tend to show less sensitivity to negative outcomes and therefore tend to make risky choices (Wu et al., 2009). The effect of PD medication (dopamine agonist) in enhancing risk-seeking tendency was also confirmed using the Balloon Analog Risk Task—an elegant assay for risk-related behavior (Claassen et al., 2011). The impairment of risk-processing in PD patients (Ehrsson et al., 2003; Wu et al., 2009; Claassen et al., 2011), and altered SM in PD, makes a risk-based motor control approach to PG performance even more compelling.

None of the previously explained computational models for PG lift tasks (Kim and Inooka, 1994; Fagergren et al., 2003; de Gruijl et al., 2009) explain the grip force levels used by controls and PD patients. Hence, a computational model to explain grip forces in controls and PD forms the motivation for the present work.

Drawing from the aforementioned presentation of facts, we propose to model PG performance, and its alterations in PD condition, using the mathematics of risk. We use the concept of utility function, a combination of value and risk components, embedded in the framework of Reinforcement Learning (RL) (Bell, 1995; Long et al., 2009; Wu et al., 2009; Wolpert and Landy, 2012). Concepts from RL have been used extensively in the past to model the function of the Basal Ganglia (BG) in control and PD conditions (Sridharan et al., 2006; Gangadhar et al., 2008, 2009; Chakravarthy et al., 2010; Krishnan et al., 2011; Magdoom et al., 2011; Kalva et al., 2012; Pragathi Priyadharsini et al., 2012; Sukumar et al., 2012). In a recent modeling study, we used the utility function to model the role of the BG in reward, punishment and risk based learning (Pragathi Priyadharsini et al., 2012).

We now present a computational model for human PG performance in controls and PD subjects in (ON/OFF) medicated states. Using risk-based DM to model PG performance, we show the alteration of *F*_*G*_ in PD patients (Wolpert and Landy, 2012) in a modified RL framework. Modeling results match favorably with experimental PG performance.

The paper is organized as follows. Section “Model” presents the model. Section “The Precision Grip Control System” presents PG control system. Section “The Utility Function Formulation and Computing *U*(*F*_Gref_)” presents the utility function formulation and Section “Modeling Precision Grip Performance as Risk-Based Action selection” presents a model of the BG based on the same (Magdoom et al., 2011; Pragathi Priyadharsini et al., 2012; Sukumar et al., 2012). In the results section, the model of the BG is used to explain PG performance of PD patients described by Ingvarsson et al. (1997) and Fellows et al. (1998). A discussion of the proposed model and modeling results is presented in the final section.

## Model

### The complete proposed precision grip model in a nutshell

1. We first define a closed-loop control system in which the plant (the finger and object system) is controlled by two controllers—a *F*_*L*_ controller and a *F*_*G*_. There are two inputs to the entire loop—a reference grip force (*F*_Gref_) and a reference position (*X*_ref_). The reference position, the position to which the object must be lifted, is predefined for a given task by the experimenter. We are now left with *F*_Gref_ as the crucial parameter that determines the PG performance of the control system. *F*_Gref_ is given as a step input to the *F*_*G*_ controller; the output of the controller, *F*_*G*_(*T*), is used to grip the object (‘*T*’ denotes simulation time in *milliseconds*). The challenge consists of finding *F*_Gref_ that leads to successful lifts.
2. We then construct the Utility function, *U*(*F*_Gref_), consisting of both value and risk components, as a function of *F*_Gref_ using a modified RL approach. The problem of finding the optimal *F*_Gref_ is then treated as an action selection problem in the BG. In previous studies, we introduced the Go-Explore-NoGo (GEN) (Magdoom et al., 2011; Sukumar et al., 2012) method as a model of action selection in the BG. In the present study, we apply GEN on *U*(*F*_Gref_) to simulate PG performance results for control and PD condition.
3. Using the gradient of the Utility function, δ_*U*_ (representing the dopamine signal), as a key signal in control of action selection, we simulate the PG performances of controls and PD subjects in the experimental tasks by Ingvarsson et al. (1997) and Fellows et al. (1998).

### The precision grip control system

**Figure 1:**
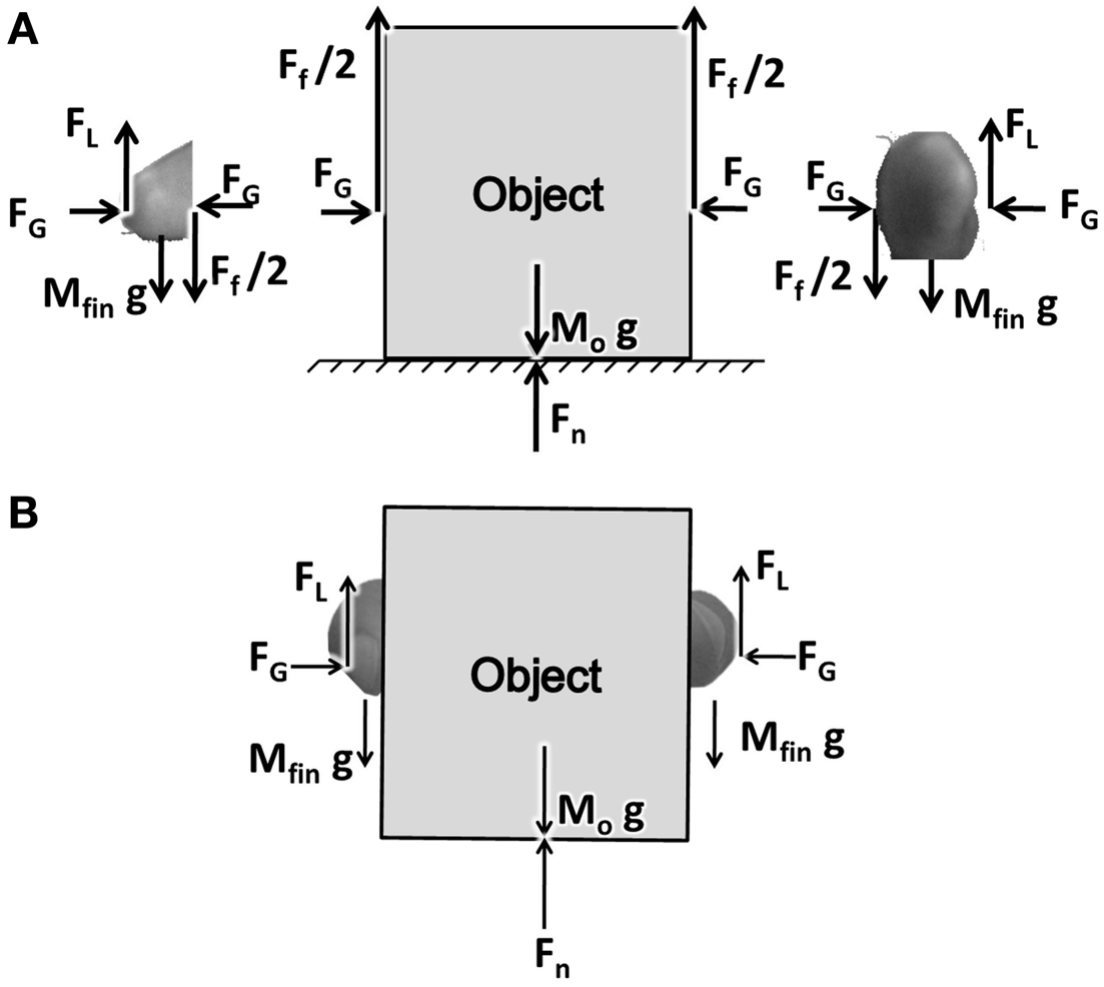
**(A)** A free body diagram showing the forces acting on object and finger. *F*_*G*_, *F*_*L*_, *F*_*f*_, *F*_*n*_ stands for Grip, Lift, frictional, and normal forces, respectively. **(B)** The Figure showing the coupling between the finger and the object.

PG performance consists of two fingers and an object interacting through friction (represented by friction coefficient μ). A free body diagram showing the various forces acting on the fingers and the object are shown in Figure 1. The fingers, shown in two parts on either side of the object, represent the index finger and the thumb. For simplicity we assume that the two fingers are identical in mass and shape.

*F*_*G*_ is the grip force applied on the object horizontally acting in opposite directions. *F*_*L*_ is the lift force acting at the finger-object interface, lifting the object up. By the action of the *F*_*G*_ pressing on the object, and due to the friction between the finger and object, *F*_*L*_ gets coupled to the object. The forces thus emerging between the finger and object are shown in Figure 1B. The frictional force *F*_*f*_ acts on the object in the upward direction, with *F*_*f*_/2 on either side of the object.

**Figure 2:**
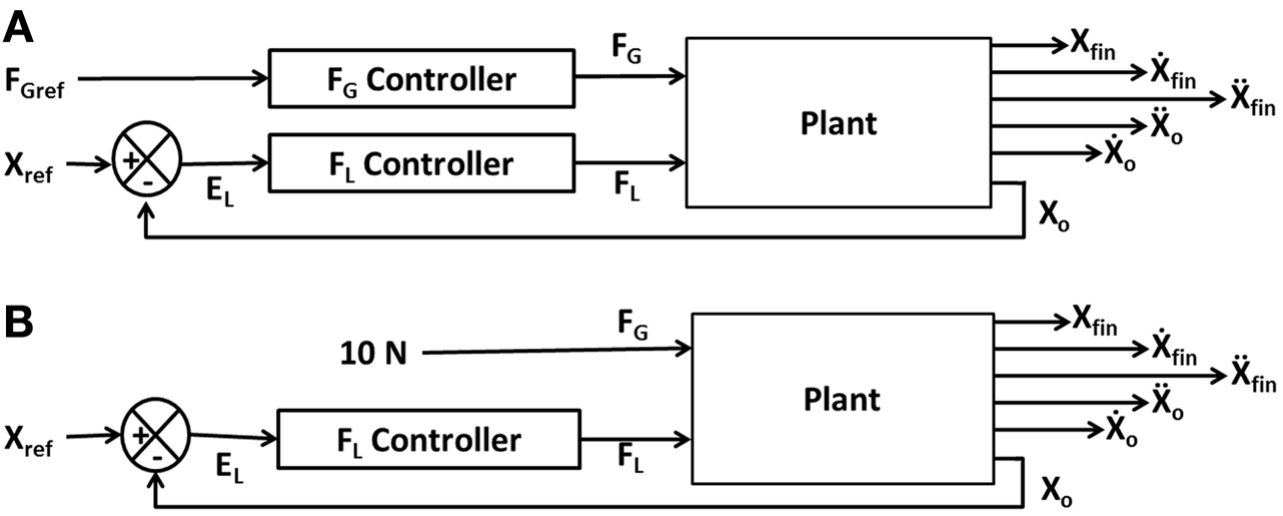
**Block diagram showing (A) the interaction of the various components and their corresponding inputs and outputs**. *X*, *Ẋ*, and *Ẍ* are the position, velocity, and acceleration; subscript “fin” and “o” denote finger and object, respectively; **(B)** the control loop used for *F*_*L*_ controller design. The grip force in the full system of panel **(A)** is set to a constant value of 10N.

Figure 2A illustrates the interaction of *F*_*L*_ and *F*_*G*_ in controlling the position of the object (*X*_*o*_). The model consists of two controllers (*F*_*L*_ and *F*_*G*_ controllers) and a plant. Inputs to the plant are *F*_*L*_ and *F*_*G*_ while the outputs are the object position (*X*_*o*_), finger position (*X*_fin_) and their derivatives (*Ẋ*_*o*_, *Ẍ*_*o*_, *Ẋ*_fin_, *Ẍ*_fin_). The objective of the control task is to lift the object to a reference position, *X*_ref_. The model receives *F*_Gref_ and *X*_ref_ as the inputs and the *X*_fin_ (position of finger), *X*_*o*_ (position of object), *Ẋ*_fin_ (velocity of finger), *Ẋ*_*o*_ (velocity of object), *Ẍ*_fin_ (acceleration of finger) and *Ẍ*_*o*_ (acceleration of object) as outputs. The plant is described in detail in Appendix A.

The following sections describe the design of the controllers (*F*_*G*_ and *F*_*L*_) followed by their training method, respectively.

#### The grip force (*F_G_*) controller

The *F*_*G*_ controller (designed as a second order system) is used to generate the *F*_*G*_ which couples the fingers to the object. Typical *F*_*G*_ profiles of human subjects show a peak and a return to a steady state value, resembling the step-response of an underdamped second order system, thereby justifying the choice of an underdamped second order system as a minimal model. The *F*_*G*_ controller for a step input (*F*_Gref_) is given in Equation (1).

(1) $$F_{G}=\frac{\omega_{\text{n}}{}^{\text{2}}}{\left({\text{s}}^{\text{2}}+2\omega_{\text{n}}\;\zeta \text{s}+\omega_{\text{n}}{}^{\text{2}}\right)}$$

In order to determine the values of natural frequency, ω_*n*_ and damping factor, ζ, maximum overshoot (*M*_*p*_, defined as the maximum peak value of the response curve) and time to peak (*T*_*p*_, peaking time of the response curve) are required. Using prior published experimental values (Johansson and Westling, 1984) for *M*_*p*_ and *T_p_*, *F*_*G*_ controller parameters are obtained using Equations (2, 3) (Ogata, 2002).

(2) $$M_{p}=e^{-\left(\zeta \omega_{n}/\omega_{d}\right)\pi }$$

(3) $$T_{p}=\frac{\pi }{\omega_{\text{d}}}$$

where ω_*d*_ the damped natural frequency is given as,

(4) $$\omega_{d}=\omega_{n}\sqrt{1-\zeta^{2}}$$

See “Controller Training from the Model of The Precision Grip Control System” for the details of the above calculations.

#### Lift force (*F*_*L*_) controller

The *F*_*L*_ controller, which is a Proportional-Integral-Derivative (PID) controller [Equation (5)], takes the position (*E*_*L*_) as input [Equation (6)], and produces a time-varying *F*_*L*_ profile (*F*_*L*,PID_) as output [Equation (5)] which in turn controls the object position.

(5) $$F_{L,\text{ PID}}=K_{P,L}E_{L}+K_{I,L}\int_{0}^{T}E_{L}(\tau_{n})d\tau_{n}+K_{D,L}\frac{dE_{L}}{dT}$$

(6) $$E_{L}=X_{\text{o}}-X_{\text{ref}}$$

Here the *K_P,L_*, *K_I,L_* and *K_D,L_* are the proportional, integral and derivative gains, respectively, for the *F*_*L*_ controller.

PID controller output is non-zero initially which is not realistic since the initial value of *F*_*L*_must be zero. Hence, *F*_*L*, PID_ is smoothened using Equation (7).

(7) $$\tau_{s}\;\frac{dF_{L}}{dT}=-F_{L}+F_{L,\text{PID}}$$

where *F*_*L*_ (*T* = 0) = 0.

In order to design the *F*_*L*_ controller, we simplify the full system of Figure 2A as a *F*_*L*_ controller with a high constant *F*_*G*_ (10 N) to prevent the slip (Figure 2B). Note that if a constant and high value of *F*_*G*_ is assumed, slip is completely prevented, and the *F*_*G*_ controller is effectively eliminated from the complete system (Figure 2B). The *F*_*L*_ controller design now involves lifting a simple inertial load straight up from an initial position (*X*_*o*_ = 0 m) to a final position (*X*_*o*_ = 0.05 m).

Performance of the lift is evaluated using the cost function, CE, [Equation (8)]. This Cost function comprises of (1) average position difference between the finger (*X*_fin_) and the object (*X*_*o*_) at the end of the trial and (2) the difference in position between the desired and actual average position of the object.

(8) $$CE\;({\text{F}}_{\text{Gref}})=0.5{\left(\frac{{\bar{X}}_{\text{fin}}-{\bar{X}}_{o}}{{\bar{X}}_{\text{fin}}}\right)}^{2}+0.5{\left(\frac{X_{\text{ref}}-{\bar{X}}_{o}}{X_{\text{ref}}}\right)}^{2}$$

**Figure 3:**
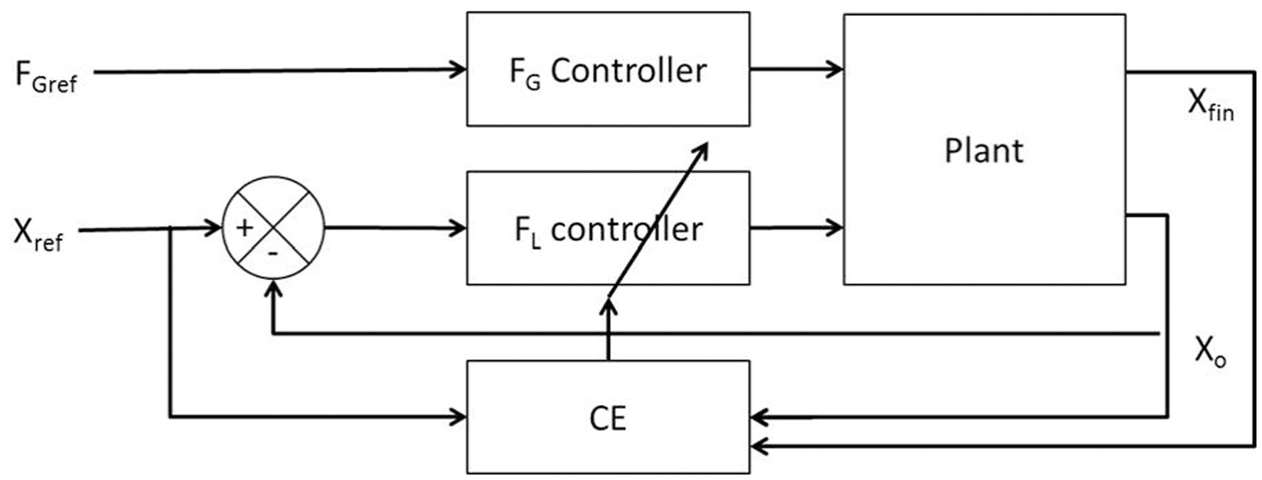
**Block diagram showing the training mechanism of the *F*_*L*_ controller**.

The *F*_*L*_ PID controller parameters were then optimized for cost function (CE) using Genetic Algorithm (GA) (Goldberg, 1989; Whitley, 1994) (refer Figure 3 for block diagram and for parameters refer Supplementary Material A) keeping *F*_*G*_ constant (=10 N) at a sufficiently high value so that the object does not slip.

The *F*_*L*_ controller described above is designed assuming a constant and high grip force. But, when both *F*_*G*_ and *F*_*L*_ controllers are inserted in the full system (Figure 2A) the system may behave in a very different manner due to gradual *F*_*G*_ buildup starting from zero. When a step input of magnitude *F*_Gref_ is given to the *F*_*G*_ controller, the *F*_*G*_ starts from 0, then approaches a peak value and stabilizes at a steady-state value known as Stable Grip Force (*SGF*). Even with the trained controllers, for low values of *F*_Gref_, the object may slip. But once the *F*_Gref_ is sufficiently high, slip is prevented and the object can be lifted successfully. Therefore, for a successful lift, an optimal value of *F*_Gref_ needs to be determined. The optimal *F*_Gref_, which is related to *SGF* needed for a successful grip/lift performance, varies with the experimental setup, skin friction etc. (Ingvarsson et al., 1997; Fellows et al., 1998). It is here that we use concepts from RL and the utility function formulation for searching the *F*_Gref_ state space.

### The utility function formulation

The optimality of a decision is measured by the rewards fetched by it. Selection of an optimal choice providing the maximum expected value of the rewards (value) is known as optimal decision making (DM). DM can be seen in stock exchange, corporates and even our daily lives (which may or may not involve explicit monetary rewards). A lot of DM is carried out subconsciously when the person is unaware of the reason for choosing ones decision (Ferber et al., 1967). Non-human primates also show DM capabilities (Lakshminarayanan et al., 2011; Leathers and Olson, 2012). A mathematical framework is essential to empirically understand subjective DM behavior. Various independent studies confirm the role of value (average reward) and risk (variance in the rewards) in DM (Milton and Savage, 1948; Markowitz, 1952; Hanoch and Levy, 1969; Kahneman and Tversky, 1979; Bell, 1995; Lakshminarayanan et al., 2011).

A search for components of DM in the living world lead to identification neural correlates of value and risk in human and non-human primates (D'Acremont et al., 2009; Schultz, 2010; Lakshminarayanan et al., 2011; Leathers and Olson, 2012). Human DM process takes account of the risk in addition to the mean rewards that the decisions fetch. Furthermore, many neurobiological correlates of risk sensitivity are found in support of risk-based DM in humans (Wu et al., 2009; Schultz, 2010; Zhang et al., 2010; Wolpert and Landy, 2012).

An important instance of risk-based DM model is the utility function formulation, which is a combination of value and risk information (D'Acremont et al., 2009). The utility function used in the current model of PG performance derives from (Bell, 1995; D'Acremont et al., 2009) study that uses the concept of utility (*U*) as a weighted sum of value (*V*), which represents expected reward, and risk (*h*), which denotes variance in the reward. The weighting factor, λ, involved in the linear sum of *V* and *h*, denotes risk preference [Equation (9)].

(9) $$U=V-\lambda \sqrt{h}$$

We here introduce few terms from RL used in our study, following a policy (π) is associated with an expected value (*V*) of the state (s) at trial (*t*) is given by Equation (10).

(10) $$V(t)=E_{\pi }\left(r(t+1)+\gamma \;r(t+2)+\gamma^{2}\;r(t+3)+\ldots |s(t)=s\right)$$

where, E is expectation and γ is the discount factor denoting the myopicity in the prediction of future rewards, and the reward is denoted by “*r*.” The *V* update is by Equation (11).

(11) $$V(t+1)=V(t)+\eta_{V}\delta (t)$$

where η_*V*_ is the learning rate for *V* and “δ ” is the temporal difference error or the reward prediction error, and is given by Equation (12)

(12) δ(t)=r(t+1)+γ V(t+1)−V(t)

Similarly, the risk prediction error, “ξ(*t*)” is denoted by Equation (13).

(13) $$\xi (t)=\delta {\left(t\right)}^{2}-h(t)$$

where, the risk function denoted by (*h*) is the variance in the prediction error [Equation (14)]

(14) $$h(t+1)=h(t)+\eta_{\text{risk}}\;\xi (t)$$

Here η_risk_ is the learning rate for risk. We now introduce a modified version of Utility (*U*) [Equation (9)], as follows,

(15) $$U(t)=V(t)-\alpha \text{ sign}(V(t))\;\sqrt{h(t)}$$

where “α sign(*V*(*t*))” is the risk preference (Pragathi Priyadharsini et al., 2012).

The sign(*V*) term achieves a familiar feature of human decision making viz., risk-aversion for gains and risk-seeking for losses (Kahneman and Tversky, 1979). In other words, when *V* is positive (negative), *U* is maximized (minimized) by minimizing (maximizing) risk. We now use the above basic framework for modeling the Utility as a function of *F*_Gref_.

### Computing U(*F*_Gref_)

We now explain how the above-described Utility function formulation is applied in the present study. The Utility function, *U*, and its components *V* and *h*, are expressed as functions of *F*_Gref_, which is taken as the state variable. A given value of *F*_Gref_ results in either slip or successful lift of an object. The measure of performance is expressed by CE [Equation (8)].

The value “*V*” and risk “*h*” are computed as a function of *F*_Gref_ by repeatedly simulating lift for a range of values around *F*_Gref_. This helps us to analyze the possible variability caused on the performance of the PG task with *F*_Gref_. A range of values were obtained by adding uniformly distributed noise (ν : refer Table 1) to *F*_Gref_ [Equation (16)]. We modeled ν to proportionally represent the *F*_slip_ i.e., the value of ν is increased for a low μ.

(16) $${\hat{F}}_{\text{Gref}}=F_{\text{Gref}}+\nu$$

**Table 1:** **Parameters used in simulation: Mass of the object (*M*_o_), friction coefficient (μ) and noise added (ν) for different cases simulated in the study**.

	**Ingvarsson et al. (1997) silk**	**Ingvarsson et al. (1997) sandpaper**	**Fellows et al. (1998)**
*M_o_* (in kg)	0.3	0.3	0.33
μ	0.44	0.94	0.44
ν	Uniformly distributed ∈ [−3,3] N	Uniformly distributed ∈ [−1.5,1.5] N	Uniformly distributed ∈ [−3,3] N
Conditions simulated	Controls, PD OFF, PD ON	Controls, PD OFF, PD ON	Controls, PD ON

**Figure 4:**
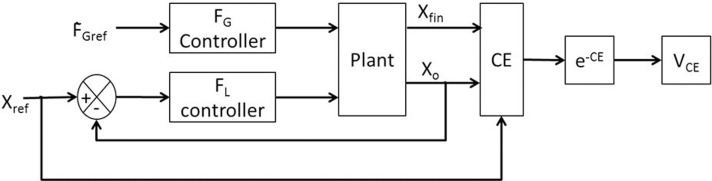
**Block diagram showing the generation of error function (*V*_*CE*_) for the input *F*_Gref_**.

For the given value of *F*_Gref_, *V*_*CE*_ was drawn from the performance measure [Equation (8)] and is generated using the cost function CE as [Equation (17)]. Refer Figure 4 for the block diagram for determining *V*_*CE*_.

(17) $$V_{CE}\left({\hat{F}}_{\text{Gref}}\right)=e^{-CE\left({\hat{F}}_{\text{Gref}}\right)}$$

There is no explicit reward in the present formulation. *V*_*CE*_ represents a reward-like quantity, the average of which is linked to value. Value [Equation (18)] and risk [Equation (19)] were calculated as the mean and variance of the *V*_*CE*_,

(18) $$V\left(F_{\text{Gref}}\right)=\text{mean }\left(V_{CE}\left({\hat{F}}_{\text{Gref}}\right)\right)$$

(19) $$h\left(F_{\text{Gref}}\right)=\text{var}\left(V_{CE}\left({\hat{F}}_{\text{Gref}}\right)\right)$$

We now have a set of numerical values of *F*_Gref_ and the corresponding *V* and *h* values. These numerical values are used to construct *V* and *h* as explicit functions of *F*_Gref_ using Radial Basis Function Neural Networks (RBFNNs) explained in Appendix B. The above mentioned Equations (18, 19) is general to all the trials. These processes are summarized in Table 2.

**Table 2:** **Steps to train value = *V*(*F*_Gref_) and risk = *h*(*F*_Gref_)**.

•	*F*_Gref_ is randomly chosen between [1N 10N].
•	Choose noise “ν ” (uniformly distributed in [−1.5,1.5] N for μ = 0.94, and [−3,3] N for μ = 0.44).
•	Calculate ${\hat{F}}_{\text{Gref}}$ as ${\hat{F}}_{\text{Gref}}$ = F_Gref_+ ν [Equation (16)] at a particular trial, t.
•	Obtain error *CE*[${\hat{F}}_{\text{Gref}}$(*t*)] using Equation (8) for 2500 instances of simulated precision grip (model of section “The Precision Grip Control System”) performance using ${\hat{F}}_{\text{Gref}.}$
•	Calculate *V*[*F*_Gref_(*t*)] and *h*[*F*_Gref_(*t*)] using Equations (18) and (19), respectively.
•	Train two separate RBFNNs to generate V[*F*_Gref_(*t*)] and *h*[*F*_Gref_(*t*)] with *F*_Gref_ as input (see Appendix B for details).
•	Utility is calculated using Equation (20).

Specifically, for a *F*_Gref_ chosen at trial, *t*, *U*(*F*_Gref_(*t*)) is a combination of the *V*(*F*_Gref_(*t*)) and *h*(*F*_Gref_(*t*)). Utility, *U*(*F*_Gref_(*t*)) [Equation (20)], is then obtained in terms of *V* and *h*, as shown below:

(20) $$U\left(F_{\text{Gref}}(\text{t})\right)=V\left(F_{\text{Gref}}(\text{t})\right)-\alpha \;\sqrt{h\left(F_{\text{Gref}}(\text{t})\right)}$$

Decisions are made by choosing actions that maximize *U*(*F*_Gref_(*t*)). Increasing the value of α makes the decision more risk aversive, while the decisions are more risk-seeking for smaller values of α. Maximizing *U*(*F*_Gref_(*t*)) is done by a stochastic hill-climbing process called the “Go/Explore/NoGo” (GEN) method. This method is inspired by dynamics of the BG and was described in greater detail in earlier work (Magdoom et al., 2011). We now present a brief account of the GEN method.

### Modeling precision grip performance as risk-based action selection

The key underlying idea of the proposed model is to treat the problem of choosing the right *F*_*G*_ as an action selection problem and thereby suggest a link between the action selection function of the BG and PG performance. Impairment in action selection machinery of the BG under PD conditions is then invoked to explain *F*_*G*_ changes in PD ON and OFF conditions.

In line with the tradition of Actor-Critic approach to modeling the BG (Joel et al., 2002); we recently proposed a model of the BG in which value is thought to be computed in the striatum. Furthermore, we hypothesized that the action selection function of the Basal Ganglia is accomplished by performing some sort of a stochastic hill-climbing over the value function computed in the striatum. We dubbed this stochastic hill-climbing method the “Go/Explore/NoGo” (GEN) method (Magdoom et al., 2011; Kalva et al., 2012), which denotes a conceptual expansion of the classical Go/NoGo picture of the BG function. As an extension of the above model, we had recently proposed a model of the BG in which the striatum computes not just value but the Utility function (Pragathi Priyadharsini et al., 2012). Action selection is then achieved by applying the GEN method to the Utility function. In the present study, we apply the GEN method to the Utility function and estimate grip forces in control and PD conditions.

#### Neurobiological interpretation of the GEN method in controls

We now present some background and neurobiological interpretation of the GEN method in connection with functional understanding of the BG, following which details of the GEN method will be provided.

**Figure 5:**
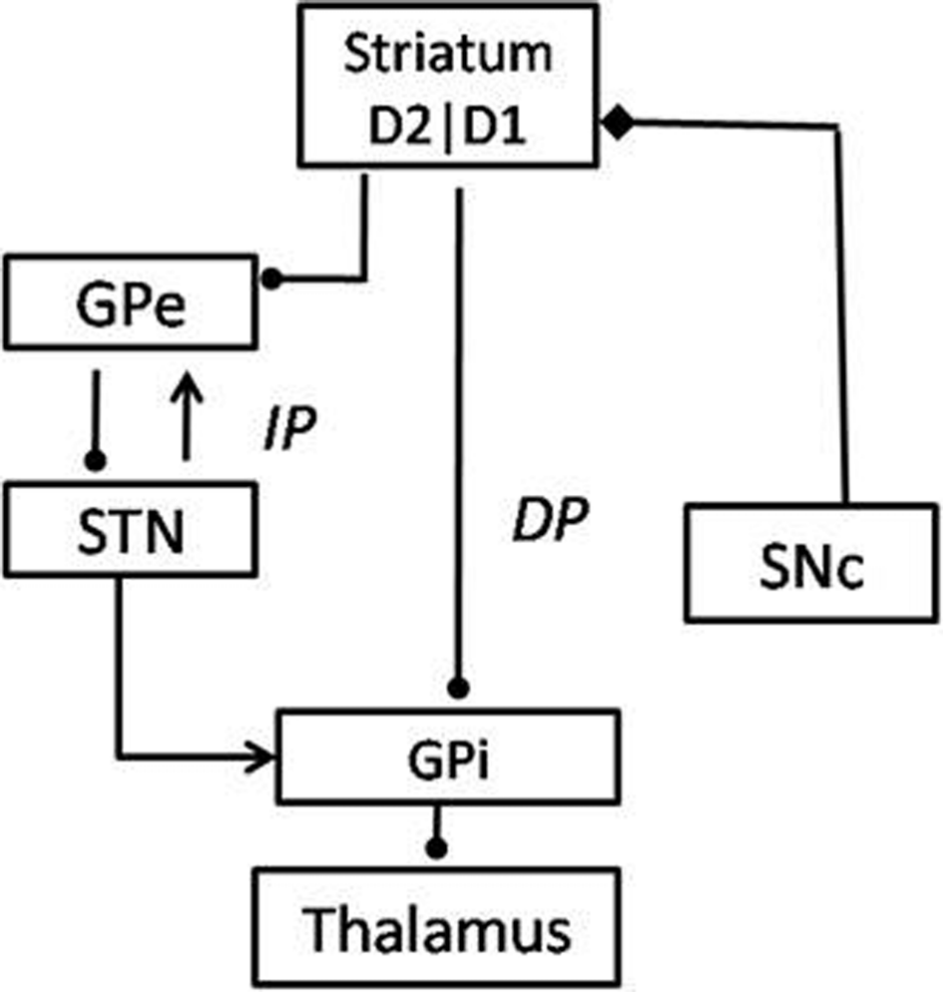
**The Basal Ganglia network with DP (Direct Pathway) and IP (Indirect Pathway) specified**.

The BG system consists of 7 nuclei situated on two parallel pathways that form loops—known as the direct pathway (DP) and the indirect pathway (IP)—starting from the cortex and returning to the cortex via the thalamus. Striatum is the input port of the BG, which receives inputs from the cortex. The Globus Pallidus interna (GPi) and the Substantia Nigra pars reticulata (SNr) are the output ports that project to thalamic nuclei, which in turn project to the cortex, closing the loop. The DP consists of the striatum projecting directly to GPi/SNr, while the IP consists of a longer route starting from the striatum and returning to GPi/SNr via Globus Pallidus externa (GPe) and the Subthalamic Nucleus (STN) in that order. The striatum receives dopaminergic projections from the Substantia Nigra pars compacta (SNc) (Mink, 1996; Smith et al., 1998). Striatal dopamine levels are thought to switch between DP and IP, since the DP (IP) is selected for higher (lower) levels of dopamine (Sutton and Barto, 1998; Frank, 2005; Wu et al., 2009) (Figure 5).

In classical accounts of the BG function, the DP is known as the Go pathway since it facilitates movement and the IP is called the NoGo pathway since it inhibits movement. Striatal dopamine levels are thought to switch between Go and NoGo regimes (Albin et al., 1989). We have been developing a view of the BG modeling in which the classical Go/NoGo picture is expanded to Go/Explore/NoGo picture, wherein a new Explore regime is inserted between the classical Go and NoGo regimes (Kalva et al., 2012). This explore regime corresponds to random exploration of action space which is a necessary ingredient of any RL machinery. Kalva et al. (2012) show that the Explore regime arises naturally due to the chaotic dynamics of the STN-GPe loop in the IP. The three regimes together amount to a stochastic hill-climbing, which we describe as the GEN method. The GEN method has been used in the past to describe a variety of functions of the BG, in control and PD conditions, including reaching movements (Magdoom et al., 2011) and spatial navigation (Sukumar et al., 2012).

Magdoom et al. (2011) used the GEN method to hill-climb over the value function (Magdoom et al., 2011). δ_*V*_(*t*) is defined as a temporal difference in value function [Equation (21)].

(21) $$\delta_{V}(t)=V(F_{\text{Gref}}(t))-V(F_{\text{Gref}}(t-1))$$

where “*t*” is not “time” but “trial number.”

The GEN method used in Magdoom et al. (2011) can be summarized using the following Equation (22),

(22) $$\begin{array}{l} \text{if}(\delta_{V}(t)>DA_{hi})\\ \begin{array}{ccc} \Delta F_{\text{Gref}}(t)=+\Delta F_{\text{Gref}}(t-1) & \;-“{\text{Go}}^{\prime \prime } & \;(\text{a}) \end{array}\\ \text{else if}(\delta_{V}(t)>DA_{lo}\wedge \delta_{V}(t)\leq DA_{hi})\\ \begin{array}{ccc} \Delta F_{\text{Gref}}(t)=\psi & \;-“\text{E}xp{\text{lore}}^{\prime \prime } & \left(\text{b}\right) \end{array}\\ \text{else}(\delta_{V}(t)\leq DA_{lo})\\ \begin{array}{ccc} \Delta F_{\text{Gref}}(t)=-\Delta F_{\text{Gref}}(t-1) & \;-“{\text{NoGo}}^{\prime \prime } & \;(\text{c}) \end{array} \end{array}$$

where, ψ is a random vector, and ||ψ|| = χ, a positive constant. *DA_hi_* and *DA_lo_* are the thresholds at which dynamics switches between Go, NoGo and Explore regimes [Equation (22)]. The underlying logic of the above set of Equations (22a–c) is as follows. If δ_*V*_(*t*) > *DA_hi_*, the last update resulted in a sufficiently large increase in *V*; therefore continue in the same direction in the next step. This case is called the “Go” (Equation 22a) regime. If δ_*V*_(*t*) ≤ *DA_lo_*, the last update resulted in a significant decrease in *V*; therefore proceed in the direction opposite to the previous step. This case is called the “NoGo” (Equation 22b) regime. If *DA*_*lo*_ < δ_*V*_(*t*) ≤ *DA_hi_*, there was neither a marked increase nor decrease in *V*; therefore Explore (Equation 22c) for new directions. This case is called the Explore regime. In Magdoom et al. (2011) we assumed a simple symmetry between *DA_hi_* and *DA_lo_*, such that *DA*_*hi*_ > 0 and *DA*_*lo*_ = −*DA_hi_*.

However, in the present study we introduce a small variation of the above formulation of the GEN method. Instead of *V*, we perform hill-climbing over the Utility landscape. The three separate Equations (22a–c) can be combined into a single Equation (23) [as in Sukumar et al. (2012)], as follows:

(23) $$\begin{array}{l} \Delta F_{\text{Gref}}(t)=A_{G}\log\text{sig}\left(\lambda_{G}\delta_{U}(t)\right)\;\Delta F_{\text{Gref}}(t-1)\;\\ \;-A_{N}\log\text{sig}(\lambda_{N}\delta_{U}(t))\;\Delta F_{\text{Gref}}(t-1)\;\\ \;+A_{E}\psi \exp(-\delta_{U}^{2}(t)/\sigma_{E}^{2}) \end{array}$$

where,

(24) $$\delta_{U}(t)=U\left(F_{\text{Gref}}(t)\right)-U\left(F_{\text{Gref}}(t-1)\right)$$

And,

(25) $$\text{logsig}(n)\;=\;\frac{1}{1\;+\text{ exp}(-n)}\;$$

Δ*F*_Gref_ is the change in *F*_Gref_ and ‘*t*’ is the trial number; *A*_*G/E/N*_ are the gains of Go/Explore/NoGo components, respectively; λ_*G/N*_ are the sensitivities of the Go/NoGo components, respectively; ψ is a random variable uniformly distributed between −1 and 1 and σ_*E*_ is the standard deviation used for the Explore component.

Just as TD error is interpreted as dopamine signal in classical RL-based accounts of the BG function, we interpret δ_*U*_ as dopamine signal in the present study. In Equation (23) above, the first term on the RHS corresponds to “Go” regime, since it is significant for large positive values of δ_*U*_ (since *A*_*G*_ and λ_*G*_ are positive). The second term on the RHS of Equation (23) above corresponds to “NoGo” regime, since it is significant for large negative values of δ_*U*_ (due to *A*_*N*_ > 0 and λ_*G*_ < 0). The third term on the RHS corresponds to “Explore” regime, since it is dominant for values of δ_*U*_ close to 0.

#### Neurobiological interpretation of the GEN method in PD

PD being a dopamine deficient condition, a natural way to incorporate Parkinsonian pathology is to attenuate the dopamine signal, δ_*U*_. In (Magdoom et al., 2011; Sukumar et al., 2012), PD pathology was modeled by clamping the dopamine signal, δ_*U*_, and preventing it from exceeding an upper threshold. The rationale behind such clamping is that with fewer dopaminergic neurons left, SNc may not be able to produce a signal intensity that exceeds a certain threshold. In the present case of PG, such a constraint is applied to δ_*U*_. If [a,b] is the natural unconstrained range of values of δ_*U*_ for controls, then for PD OFF simulation, a clamped value of δ_Lim_ changes the δ_*U*_ range to [a, δ_Lim_] where δ_Lim_ < b. Furthermore, to simulate the increase in dopamine levels in PD ON condition, due to administration of L-dopa, a positive constant is added to δ_*U*_, thereby changing the range of δ_*U*_ in PD ON condition to [a+ δ_Med_, δ_Lim_ + δ_Med_] [Equation (26)].

(26) $$\delta_{\text{U}}(t)=\{\begin{array}{l} {}[a,b]\;(\text{a})\text{for controls}\\ {}[a,\delta_{\text{Lim}}]\;(\text{b})\text{for PD OFF}\\ {}[a+\delta_{\text{Med}},\delta_{\text{Lim}}+\delta_{\text{Med}}]\;(\text{c})\text{for PD ON} \end{array}$$

where δ_Lim_ + δ_Med_ < *b* and *a* < *b*.

#### Training the GEN parameters

The output of the GEN system is *F*_*G*_ from which *SGF* is calculated as average *F*_*G*_ between 4000 and 5000 ms of simulation time, the mean and variance of which must match with the mean and variance of *SGF* obtained from PG experiments under control and PD conditions (Ingvarsson et al., 1997; Fellows et al., 1998). The parameters to be trained are *A*_*G/E/N*_ [gains of the Go/Explore/NoGo terms in Equation (23)], λ_*G/N*_ [sensitivity of Go/NoGo terms in Equation (23)] and σ_*E*_ [sensitivity of Explore term in Equation (23)]. Determination of the GEN parameters is done by optimizing a cost function *CE*_GEN_ given as.

(27) $$CE_{\text{GEN}}=2{\left({\bar{SGF}}_{expt}-{\bar{SGF}}_{\text{sim}}\right)}^{2}+{\left(\sigma_{expt}-\sigma_{\text{sim}}\right)}^{2}$$

*SGF* is the stable grip force generated and σ is the variance in the error. Subscripts *expt* and *sim* denote experimental and simulated values, respectively. *CE*_GEN_ is formulated such that more weightage is given to the *SGF* error and lesser to the variance in the error [Equation (27)].

**Figure 6:**
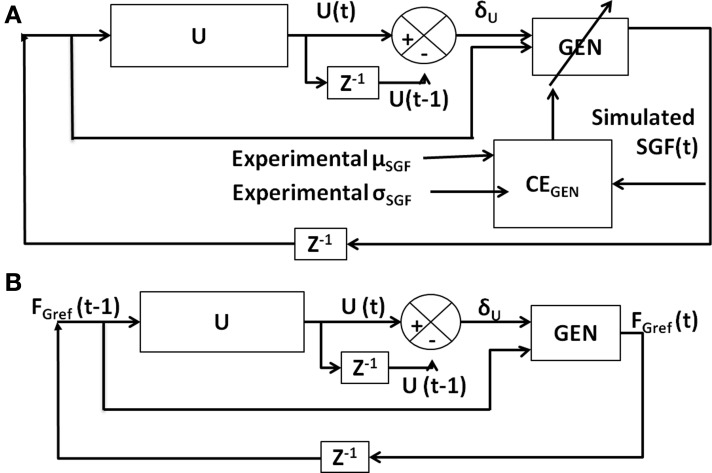
**Block diagram showing (A) training of GEN parameters using *CE*_GEN_; (B) model testing using trained GEN parameters**.

The six model parameters (*A*_*G/E/N*_, λ_*G/N*_, and σ_*E*_) of the GEN method [Equation (23)] are trained to capture the following experimental conditions: Controls and PD ON conditions are obtained from Fellows et al. (1998), whereas controls, PD ON and OFF from Ingvarsson et al. (1997) for both sandpaper and silk surfaces. However, the parameters *A*_*G/E/N*_, λ_*G/N*_, and σ_*E*_ are not all trained separately for every experimental condition. Initial parameter values for *A*_*G/E/N*_, λ_*G/N*_, σ_*E*_, and α were determined (Figure 6A) by matching the *SGF* results for controls of Fellows et al. (1998); this matching is achieved by optimizing *CE*_GEN_ [Equation (27)] using GA (Figure 6A). This initial training of GEN parameters is a kind of calibration of the parameters for a given experimental setup. Once an initial estimate of parameters was obtained, *A*_*G/E/N*_, λ_*G/N*_, and σ_*E*_ were fixed and optimal values of α, δ_Lim_, and δ_Med_ were obtained using GA for the PD conditions, of Fellows et al. (1998). Similarly, for Ingvarsson et al. (1997), the initial parameter values for *A*_*G/E/N*_, λ_*G/N*_, σ_*E*_, and α were determined by matching the *SGF* results for controls using Equation (27). For the cases of PD ON and PD OFF (Ingvarsson et al., 1997), the search space was limited to α, δ_Lim_, δ_Med_, by having fixed the values of *A*_*G/E/N*_, λ_*G/N*_, σ_*E*_ obtained from controls (Ingvarsson et al., 1997).

The model was tested (Figure 6B) using the trained GEN parameters to determine if the model generated outputs are close to the experimental values.

## Results

We now apply the model described in the previous section to explain the experimental results for two published studies (Ingvarsson et al., 1997; Fellows et al., 1998). For simplicity we used only constant weight trials (i.e., only the trials where only one load was repeatedly used for lifting). The friction coefficient was calculated as load force/slip force (Forssberg et al., 1995) [Equation (28)].

(28) $$\mu =M_{o}g/F_{\text{slip}}$$

Fellows et al. (1998) investigated 12 controls, 16 PD patients and four hemi-parkinsonian patients. All the PD subjects were in medication ON state. The subjects were asked to lift the object to a height of 4–8 cm. The study comprised of two loads 3.3 N and 7.3 N. Various combinations of these two loads were used to give rise to “light,” “heavy,” “unload,” and “load” condition. In our study, we simulated only the experimental results for the “light” condition (which featured lifting the 3.3 N object for all the trials) with a desired object lift height of 5 cm.

Ingvarsson et al. (1997) investigated the role of medication in 10 controls and 10 PD subjects under two object surface conditions (silk and sandpaper). The task required the object to be PG–lifted to a height of 5 cm above the table. The entire experiment was divided into 3 parts (a) coordination of forces, (b) adaptation to friction and (c) rapid load changes. “Coordination of forces” required the object to be lifted to 5 cm height, and maintained at that position for 4–6 s using PG. “Adaptation to friction” required the subjects to gradually reduce the grip force on the object thereby causing slip *F*_slip_ is calculated. Finally, in the “rapid load changes” task a plastic disk was dropped in a padded plate thereby causing abrupt changes in *F*_*L*_.

In the present study, we use the *F*_slip_ for determining the friction coefficient which is used to match the experimentally obtained grip force values under silk and sandpaper condition for coordination of forces case. Ingvarsson et al. (1997) reported the results in median ± Q3 quartile format. Hence for simplicity the results are assumed to be normally distributed with mean = median and Q3 = mean + 0.6745 standard deviation. The entire text reports the results in terms of mean and variance.

We now describe the simulation results starting from controller training to simulation of results from Ingvarsson et al. (1997) and Fellows et al. (1998).

### Controller training from the model of the precision grip control system

#### The *F_G_* controller

**Figure 7:**
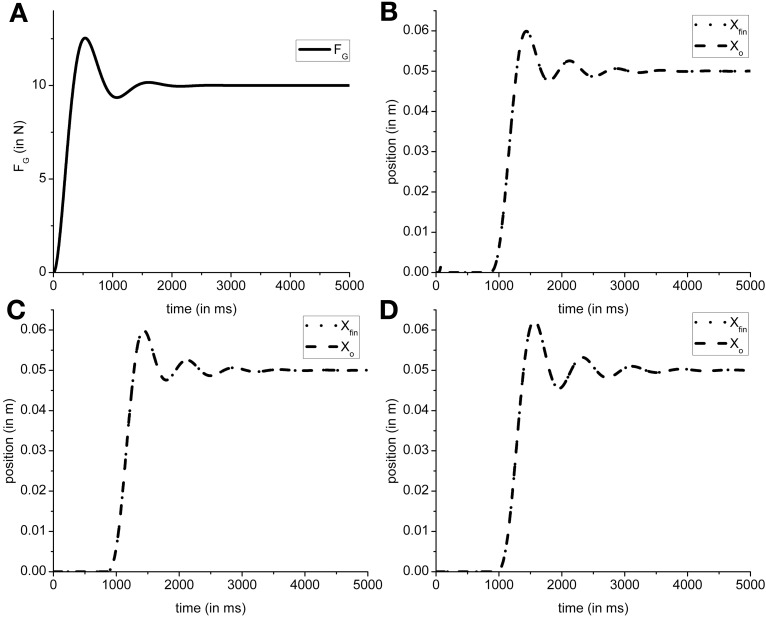
**Figure shows a single trial simulated output obtained from model. (A)** The typical *F*_*G*_ profile for the input *F*_Gref_ = 10 N. The object and finger position for **(B)** Ingvarsson et al. (1997) silk (μ = 0.44, M_*o*_ = 0.3 kg); **(C)** Ingvarsson et al. (1997) sandpaper (μ = 0.94, *M*_*o*_ = 0.3 kg); **(D)** Fellows et al. (1998) (μ = 0.44, *M*_*o*_ = 0.33 kg) are shown. Note that in generation of **(B–D)**, the *F*_Gref_ is kept constant at 10 N for illustration purpose.

In the present model the grip force controller is designed as reference tracking controller which receives *F*_Gref_ as the input and generates a time-varying *F*_*G*_(*T*) as the output. So in the proposed configuration the *F*_*G*_ controller is only affected by the input (*F*_Gref_) it receives. Using the overshoot ratio, *M*_*p*_ = 1.25, [Equation (2)] and time to peak, *T_p_* = 530 ms, [Equation (3)] as design criteria [*M*_*p*_ and *T*_*p*_ values obtained from Johansson and Westling (1984)], we determined ω_*n*_ = 6.4 and ζ = 0.4 as the parameters for transfer function of the *F*_*G*_ controller [Equation (1) in The Precision Grip Control System; Ogata, 2002]. Figure 7A shows the grip force profile (for *T* = 5000 ms) for the input *F*_Gref_ = 10 N. Since the *F*_*G*_ controller is modeled as a transfer function which is dependent only on the *F*_Gref_ value, the controller did not require retraining for different friction conditions.

#### The F_L_ controller

The efficacy of *F*_*L*_ controller output can be observed in the output object and finger position. If a suboptimal *F*_*L*_ is generated, the object does not reach the reference position. The cardinal components affecting the object-finger slip are μ and M_*o*_ (Refer Table 1 for the values of μ and M_*o*_ used in simulation). Since the *F*_*L*_ controller is affected by μ and M_*o*_, a decreased μ or increased M_*o*_ increases the *F*_slip_ Therefore, in this study we trained the *F*_*L*_ controller for the minimum μ and the maximum M_*o*_ to prevent the object from slipping even when μ increases or M_*o*_ decreases. Furthermore, to train the *F*_*G*_ controller, we assume a sufficiently large *F*_*G*_ (=10 N) thereby effectively decoupling the *F*_*G*_ controller. With a large, constant *F*_*G*_, the cost function (CE) [Equation (8)] was optimized using the GA (Goldberg, 1989; Whitley, 1994) for the setup parameters from Fellows et al. (1998) (μ = 0.44 and M_*o*_ = 0.33 N) to obtain PID parameter values. The PID parameter values obtained were *K_P,L_* = 6.938, *K_I,L_* = 14.484, *K*_*D,L*_ = 1.387, τ_*s*_ = 0.087. The same PID parameters were used to simulate results from Ingvarsson et al. (1997) also. In Figure 7, the output of the simulated finger and object position is shown for (Figure 7B) the silk condition of (Ingvarsson et al., 1997), (Figure 7C) the sandpaper condition of (Ingvarsson et al., 1997) and (Figure 7D) light condition of (Fellows et al., 1998).

Since we fixed the PID parameters for the *F*_*L*_ controller, efficacy of the PID parameters across the three control conditions viz. Ingvarsson et al. (1997) silk condition, Ingvarsson et al. (1997) sandpaper, and Fellows et al. (1998), needs to be determined. Two important criteria for determining a successful lift are: low slip (*X*_fin_ − *X*_*o*_) and low position error (*X*_ref_ − *X*_*o*_). In all the three cases (refer Table 1) the finger–object slip distance was <0.005 m and the *X*_ref_ − *X*_o_ <0.001 m, where, *X*_*o*_ is average position between simulation time (T) as 4000 and 5000 ms. The *F*_*G*_ controller output is shown in Figure 7A; object and hand position for Ingvarsson et al. (1997) sandpaper, Ingvarsson et al. (1997) sandpaper, and Fellows et al. (1998) is shown in Figures 7B–D. Note that *F*_Gref_ is held constant at 10 N.

### Obtaining *V(F*_Gref_), *H(F*_Gref_), and *U(F*_Gref_)

Utility based approach requires estimation of *V*(*F*_Gref_) and *h*(*F*_Gref_) [refer model Section “Computing *U*(*F*_Gref_)”]. *V* and *h* are generated for Ingvarsson silk and Ingvarsson sandpaper and were obtained using the parameters given in Table 1. We assumed the noise to be inversely related to the friction coefficient. Hence a higher noise was used in case of lower μ and vice versa.

Note that value functions [expressed as a function of *F*_Gref_ (Appendix B, Equation (42)] in case of PG are expected to have a sigmoidal shape, since a *F*_*G*_ level that exceeds the *F*_slip_ always results in a successful grip-lift and therefore reward. On the contrary, the risk function, *h* [Appendix B, Equation (43)], is expected to be bell-shaped since risk is the highest in the neighborhood of slip force, and zero far from it.

**Figure 8:**
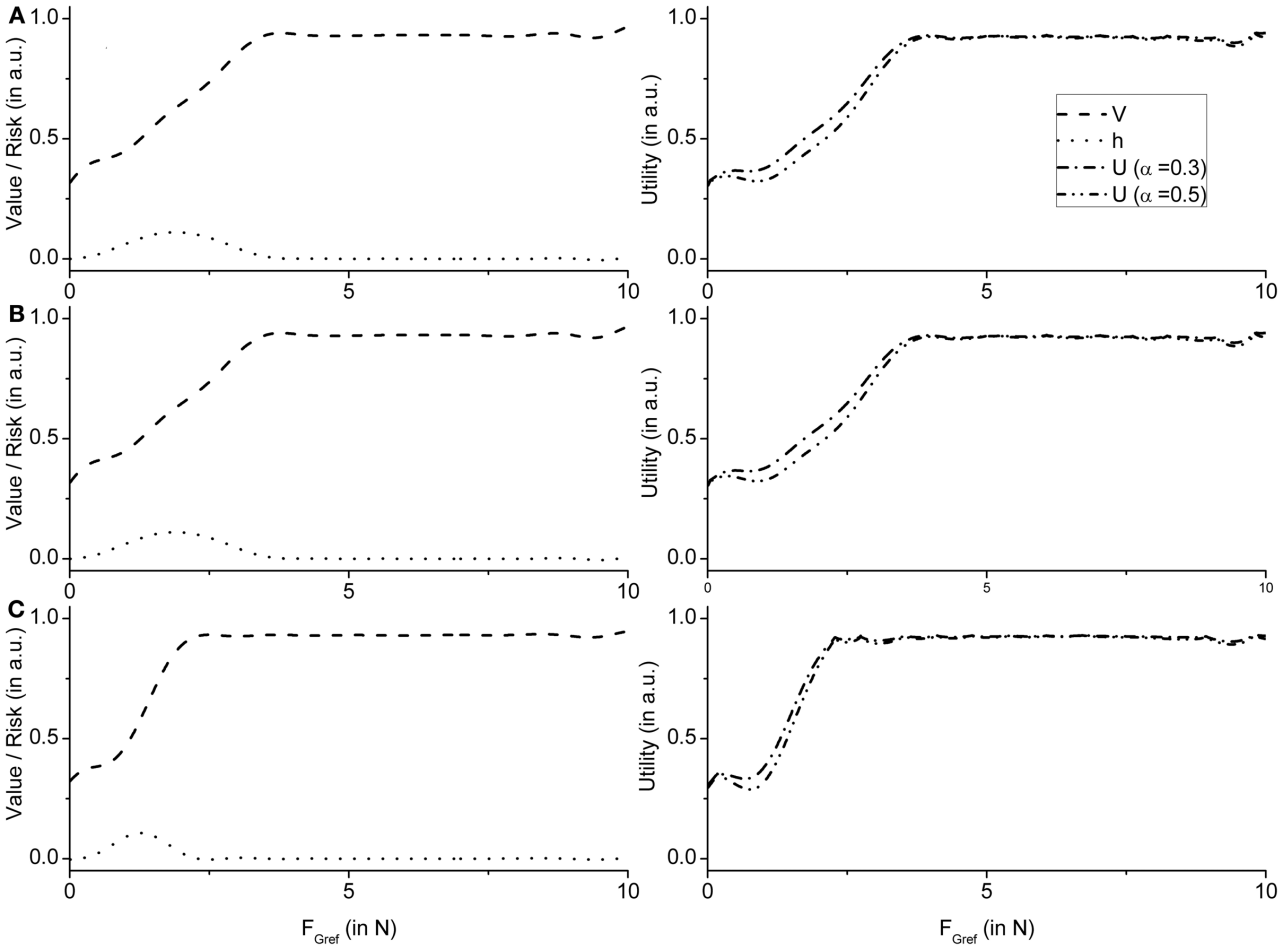
**Figure showing the value, risk and utility (α = 0.3 and α = 0.5) of the RBF network as an average value of 20 runs for **(A)** Fellows et al. (1998) **(B)** Ingvarsson et al. (1997) silk, and **(C)** Ingvarsson et al. (1997) sandpaper**.

These observations are supported by the value and risk functions constructed for various experimental conditions—Fellows et al. (1998) light and Ingvarsson et al. (1997) silk and sandpaper cases. Figure 8A shows the value and risk functions for Fellows et al. (1998), Figure 8B shows the value and risk functions for Ingvarsson et al. (1997) silk and Figure 8C shows the value and risk functions for Ingvarsson et al. (1997) sandpaper condition.

### Performing action selection using the BG and simulating the control and PD cases of the study

Two features mark the difference in *V*(*F*_Gref_) and *h*(*F*_Gref_) between controls and PD. In PD OFF case we apply the clamped δ_U_(=δ_Lim_) condition [Equation (26b)], whereas in PD ON case we add a positive constant (δ_Med_) to δ_*U*_ [Equation (26c)]. In addition to these changes in the dopamine signal, δ_*U*_, we assume altered risk sensitivity in PD. Studies also suggest altered risk taking in PD patients (in particular, risk in PD ON> risk in PD OFF) when compared to healthy controls (Cools et al., 2003). Since α represents risk sensitivity in the utility function [Equation (20)], we use a smaller α in PD case (both ON and OFF) (Refer Tables 3, 4 for the simulated values).

**Table 3:** **Table showing the GEN parameters and Utility parameters for Fellows et al. (1998) control and PD ON**.

	**Fellows et al. (1998)**	
	**Norm**	**PD ON**
λ_*G*_	1.53	1.53
λ_*N*_	−7.18	−7.18
σ_*E*_	1.00	1.00
*A_G_*	0.01	0.01
*A_N_*	1.60	1.60
*A_E_*	0.43	0.43
α	0.70	0.312
δ_Lim_	1.00	−0.5
δ_Med_	0.00	0.427

**Table 4:** **Table showing the GEN parameters and Utility parameters for simulating both Ingvarsson et al. (1997) cases of silk and sandpaper in controls, PD OFF and PD ON conditions**.

	**Ingvarsson et al. (1997)**		
	**Norm**	**PD OFF**	**PD ON**
λ_*G*_	1.53	1.53	1.53
λ_*N*_	−7.18	−7.18	−7.18
σ_*E*_	1.00	1.00	1.00
*A_G_*	0.60	0.60	0.60
*A_N_*	2.16	2.16	2.16
*A_E_*	0.29	0.29	0.29
α	0.50	0.30	0.30
δ_Lim_	1.00	0.5	0.5
δ_Med_	0.00	0.00	0.005

**Figure 9:**
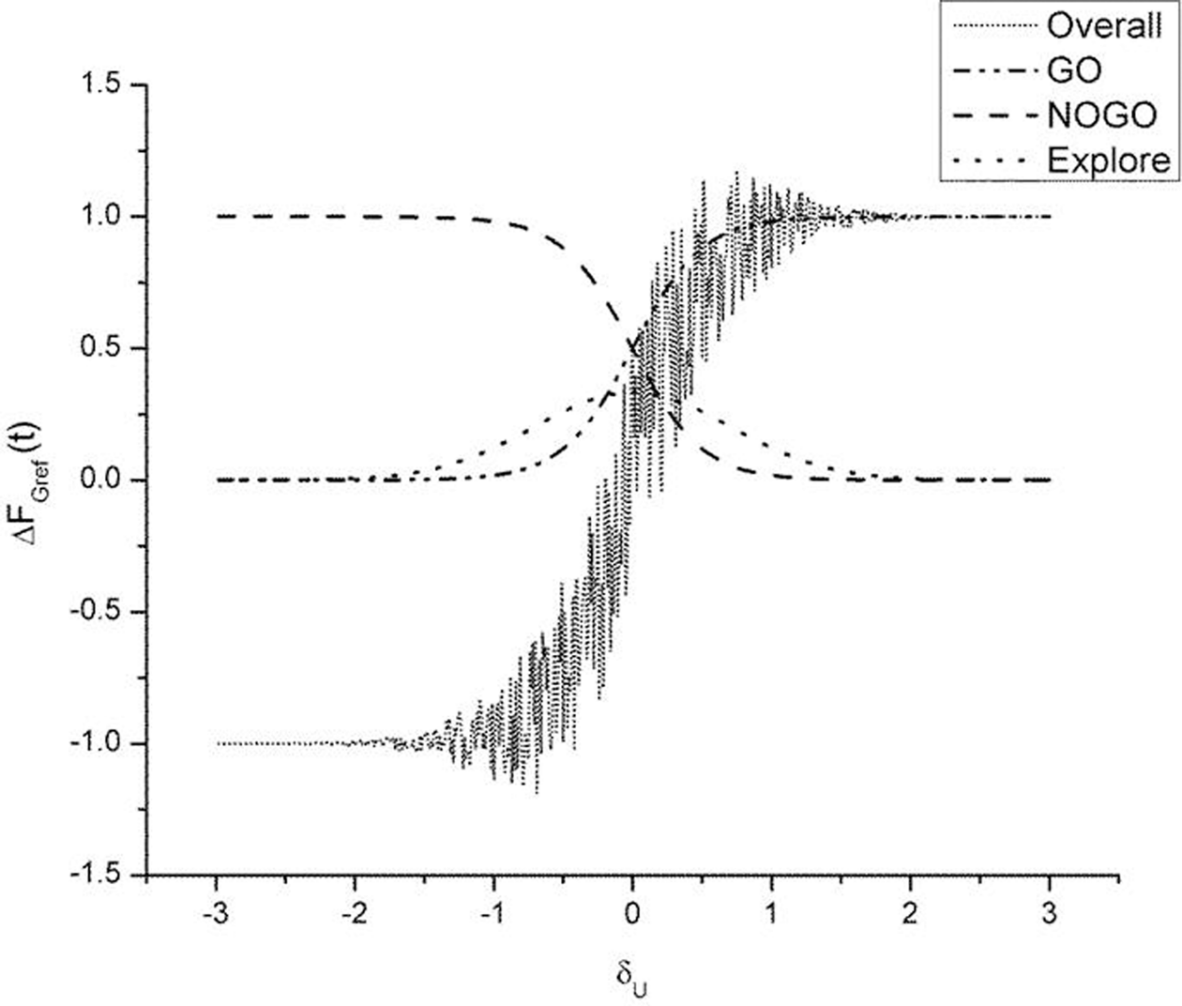
**Figure illustrating output of the GO (λ_*G*_ = 2), NOGO (λ_*N*_ = −2), explore (σ_*E*_ = 1) regime and the overall output of the three regimes**. Here *A*_*G*_ = *A_N_* = *A_E_* = 1. The Δ*F*_Gref_(*t* − 1) is set to 1. We thereby analyze the selection output Δ*F*_Gref_(*t*) as a function of δ_*U*_ alone. The “overall” graph is produced by actually adding the noise term [III term on RHS in Equation (23)]. Note the high variability in Δ*F*_Gref_(*t*) in the vicinity of δ_*U*_ = 0.

As described earlier, the GEN method [Equation (23)], produces a series of *SGF* values with a certain mean and standard deviation computed over the trials. It may be recalled, from “Model”, that Equation (23) represents a form of stochastic hill-climbing over the utility function, *U*. It represents a map from *F*_Gref_ (*t* − 1) to *F*_Gref_ (*t*), where “*t*” represents the trial number. The three terms on the RHS of Equation (23) represent GO, NOGO and Explore regimes, in that order. The three regimes are active under conditions of high, low, and moderate dopamine (δ_*U*_), respectively. Figure 9 shows some sample profiles of the three terms on the RHS of Equation (23).

Using the GEN policy on δ_*U*_, we simulate our model with parameters described in Tables 3, 4 (However, simulation with δ_*V*_ of Equation (21) with the same parameter set described in this section yields Supplementary Material B).

#### Procedure to train the GEN parameters

- The change in *F*_Gref_ (i.e., Δ*F*_Gref_) from trial, “*t*” to “*t*+1” is given in Equation (23).
- This Equation (23) contains six parameters (*A*_*G/E/N*_, λ_*G/N*_ and σ_*E*_) whose values need to be determined.
- The training for GEN parameters (*A*_*G/E/N*_, λ_*G/N*_ and σ_*E*_) was carried out using GA for optimizing *CE*_GEN_ on Fellows et al. (1998) control condition experimental data. (Figure 6A)
- The GEN parameters obtained from the control conditions are also used for simulating PD. In case of (Fellows et al., 1998), the parameters from controls are used for PD ON only. In case of (Ingvarsson et al., 1997), the parameters from controls are used in PD ON and OFF for two surface conditions – silk and sandpaper.
- In PD OFF case, only δ_Lim_ and α are trained. In PD ON case, δ_Lim_, δ_Med_ and α are trained.

#### Simulation of Fellows et al. (1998) results

Fellows et al. (1998) for controls was simulated using the parameters (Table 4) obtained by optimizing GA on *CE*_GEN._

- Using the *A*_*G/E/N*_, λ_*G/N*_, σ_*E*_ and α = 0.7 obtained from the GA, the controls was simulated using δ_Lim_ = 1 and δ_Med_ = 0.
- A similar approach was performed for modeling the PD ON condition. The parameters (*A*_*G/E/N*_, λ_*G/N*_ and σ_*E*_) were the same as the controls, while the values of α = 0.312, δ_Lim_ = −0.5 and δ_Med_ = 0.427 were obtained by GA optimization. For a detailed list of parameters see Table 3.

**Figure 10:**
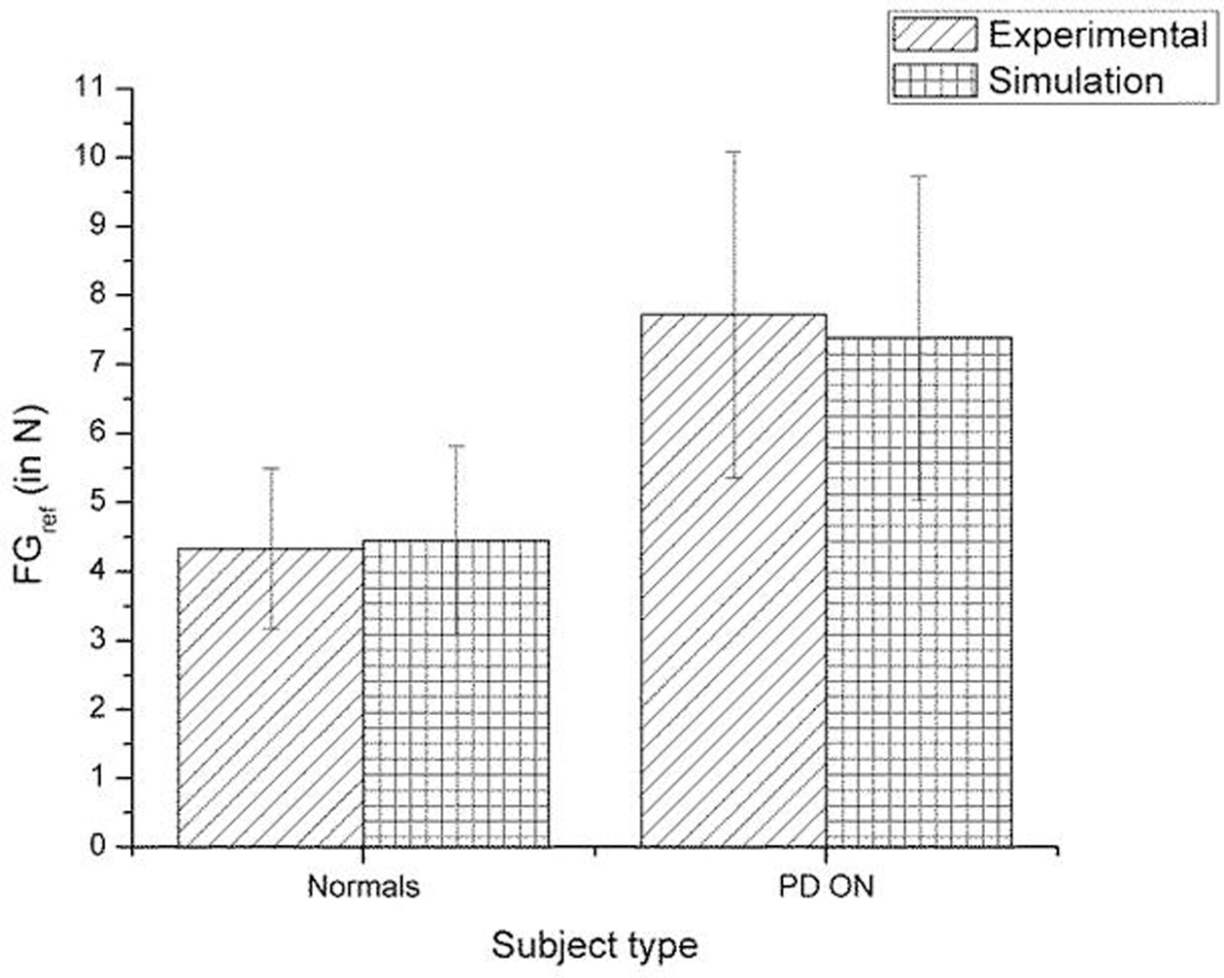
**Comparison of experimental (Fellows et al., 1998) and simulation results for *SGF***. The bars represent mean (±SEM). The results for the control and PD ON groups are statistically significant with the *p*-value < 0.05 in both the experiment and simulation.

A comparison of the experimental and simulated data obtained for Fellows et al. (1998) using the parameters in Table 3 is given as Figure 10.

#### Simulation of Ingvarsson et al. (1997) results

Following the simulation of the Fellows et al. experiment:

- The results of the Ingvarsson et al. (1997) controls for both sandpaper and silk were simulated for obtaining (*A*_*G/E/N*_, λ_*G/N*_, σ_*E*_, and α = 0.5) using GA (Table 4). The other parameters are set as δ_Lim_ = 1 and δ_Med_ = 0.
- In PD ON condition, the same control parameters (*A*_*G/E/N*_, λ_*G/N*_and σ_*E*_) were used along with α = 0.30, δ_Lim_ = 0.5 and δ_Med_ = 0.005 obtained through GA optimization.
- PD OFF condition was shown by optimizing α = 0.30, δ_Lim_ = 0.5 and δ_Med_ = 0 with the parameters (*A*_*G/E/N*_, λ_*G/N*_and σ_*E*_) remaining the same as that of the controls.

**Figure 11:**
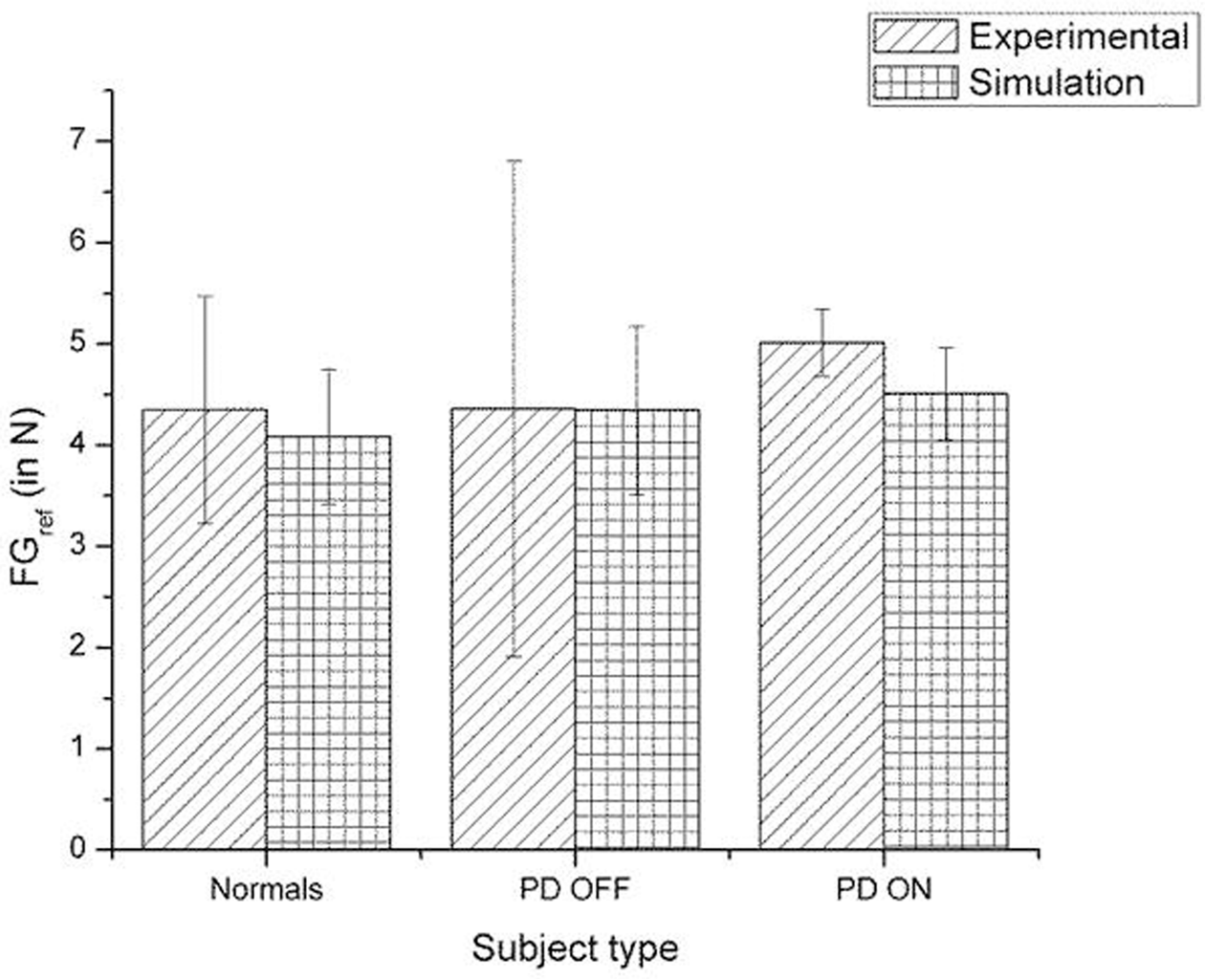
**Comparison of experimental (Ingvarsson et al., 1997) and simulation results for *SGF* for silk surface**. The bars represent the median (± Q3 quartile). The results for the control and PD ON groups are statistically significant with the *p*-value < 0.05.

**Figure 12:**
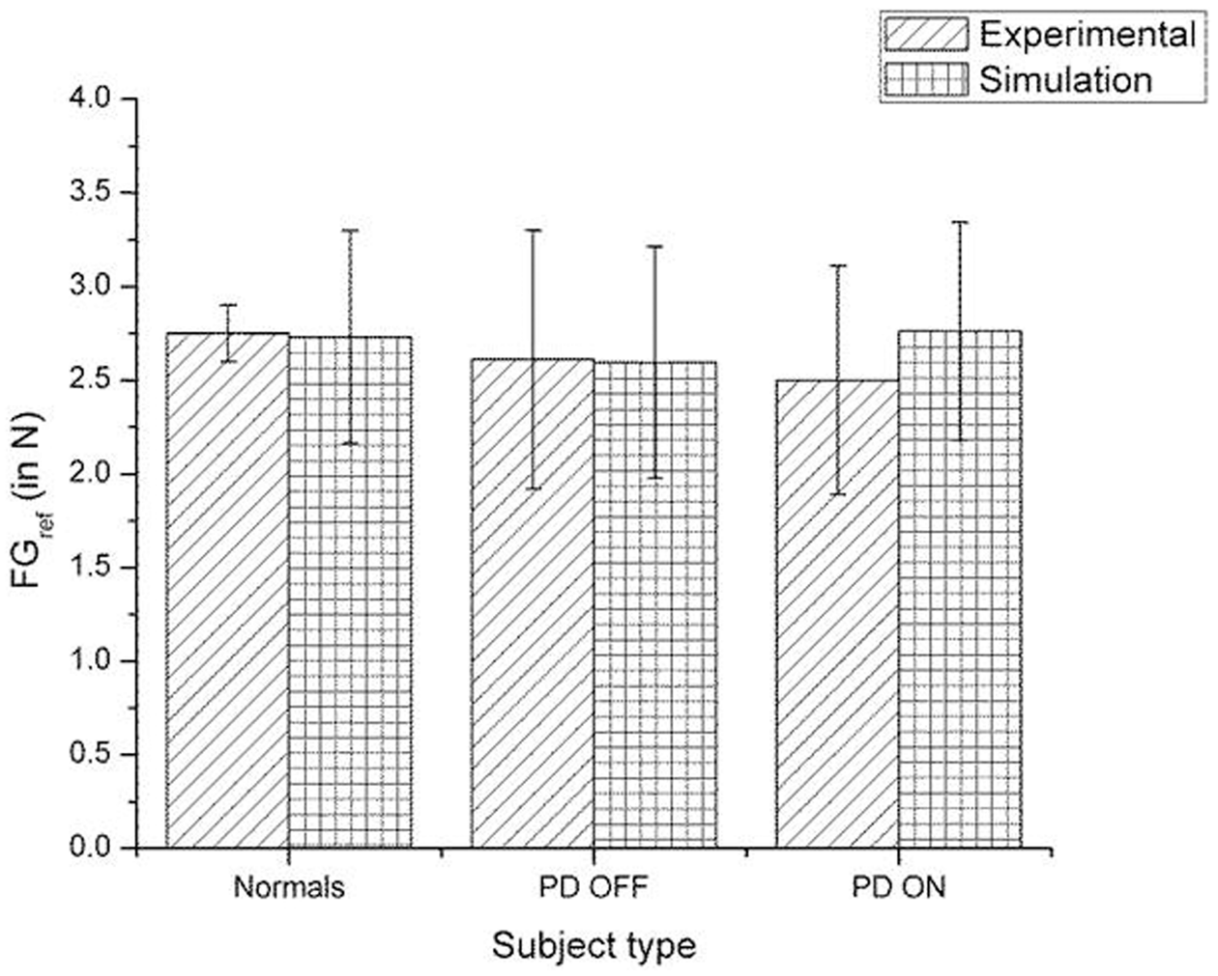
**Comparison of experimental (Ingvarsson et al., 1997) and simulation results for *SGF* for sandpaper surface**. The bars represent the median (± Q3 quartile). The results for the comparison on controls-PD ON, controls-PD OFF and PD ON-PD OFF in both the experiment and simulation are non-significant in both the experiment and simulation.

A comparison of the experimental and simulated data obtained for Ingvarsson et al. (1997) for silk and sandpaper using the parameters in Table 4 is given as Figures 11 and 12, respectively. In order to be consistent with the experimental result the simulation results in Figures 11 and 12 are shown in median ± Q3. Figures 10–12 replicate the empirical findings (both mean / median and variance profiles) well. Fellows et al. (1998) results show that the mean(*SGF*) is higher in PD ON case compared to controls (*SGF*norm<*SGF*_PDON_) (Figure 10). A similar result (*SGF*norm<*SGF*_PDON_) is also reported in Ingvarsson et al. (1997) silk (Figure 11) and sandpaper cases (Figure 12). Furthermore, var(*SGF*) under PD OFF cases is observed to be greater than that of controls. It may be inferred that the increase in *SGF* during the PD conditions would be due to their increased SM while playing risk aversive in the game of risk based decision making. This increased SM could be needed to oppose the increased internal perturbations/sensory-motor incoordination observed in the PD patients.

## Discussion

In this paper, we present a computational model to explain the changes in *F*_*G*_ in controls and PD ON/OFF conditions (Ingvarsson et al., 1997; Fellows et al., 1998). To our knowledge this is the first computational model of PG performance in PD conditions. A novel aspect of the proposed approach to modeling PG is to apply (based on the observation that PG performance involves a SM) concepts from risk-based DM to explain *F*_*G*_ generation. To this end, we applied a recent model of the BG action selection based on the utility function formulation, instead of value function, to explain PG performance (Pragathi Priyadharsini et al., 2012).

There are significant challenges involved in developing a computational model of PG in PD conditions. The root cause of this difficulty is that the pathology in PD is located at a high level (dopaminergic neurons of the BG) in motor hierarchy, while the motor expression is at the lowest level in the hierarchy (finger forces). Ideally speaking, a faithful computational model must incorporate these two well-separated levels in motor hierarchy, and all the levels in between. But development of such an extensive model would be practically infeasible, and may not be essential to the problem at hand. On the other hand, if model compactness is the sole governing principle, one may build an empirical, data-fitting model that links experimental parameters like friction, object weight, and abstract neural parameters like dopamine level, medication level, with observed data like mean and variance of grip force generated. But such an over-simplified model could be a futile mathematical exercise without much neurobiological content.

**Figure 13:**
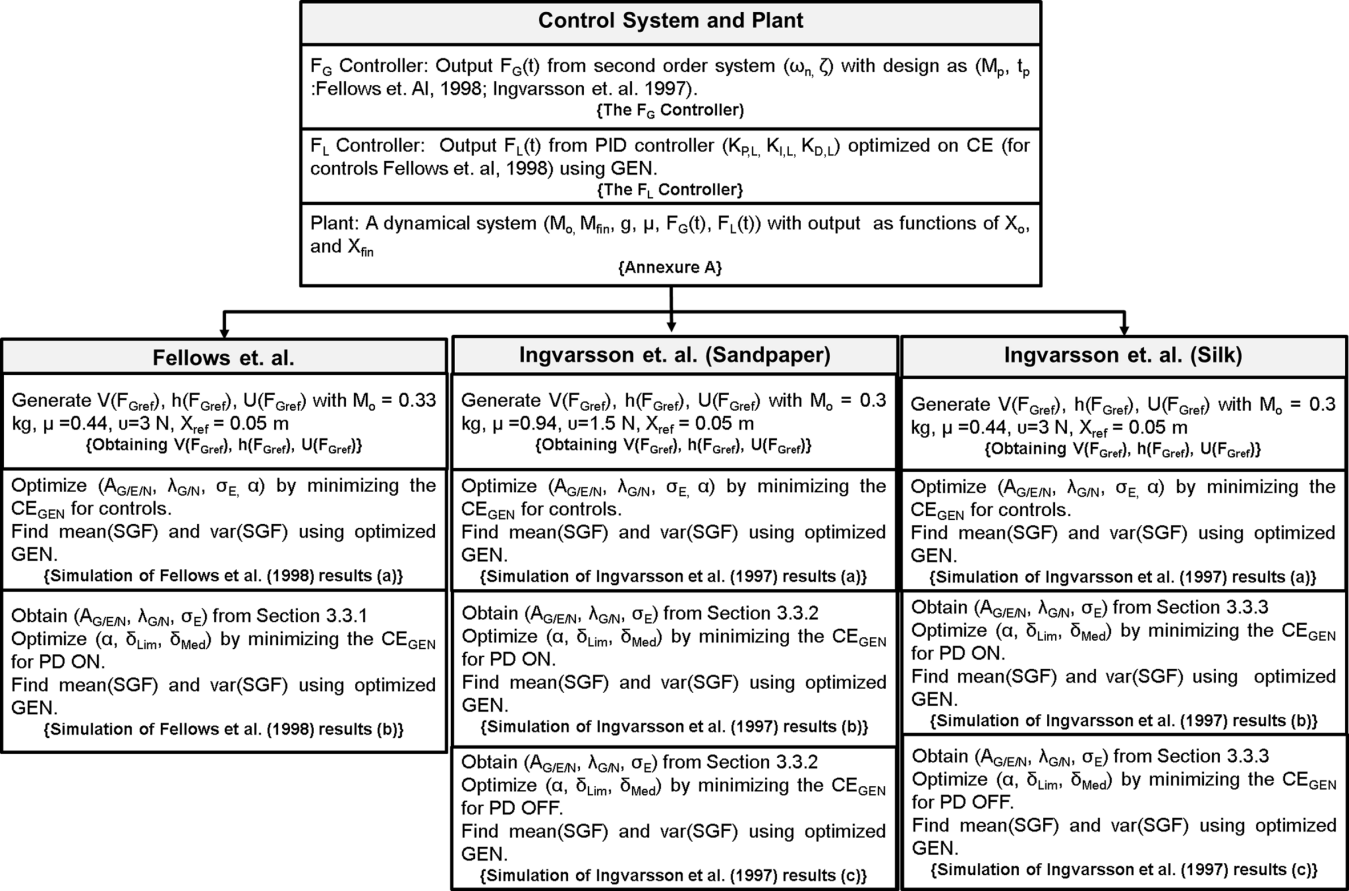
**An illustration of the entire model, the training/design steps followed to build various components and to reproduce the results of Fellows et al. (1998) and Ingvarsson et al. (1997)**. The text in {} denote section name.

Therefore, a conservative approach to model PG performance in PD would have two components: (1) a minimal model of sensory-motor loop dynamics involved in generating PG forces, and (2) a minimal model of the BG that incorporates the effect of dopamine changes on the BG dynamics. Whereas the BG level generates evaluations of actions (*F*_*G*_) based on the rewards (successful grip performance) obtained from PG performance, the sensory-motor loop dynamics receives the command from the BG level and generates *F*_*G*_. While the first component represents DM, the second represents execution. These two components must then be integrated. PD-related reduction in dopamine level in the integrated model must then be manifested as appropriate changes seen in PG forces. This is the approach adopted in the present study. Figure 13 presents the grand plan of the entire model, and the training/design steps followed to build various components.

### Modeling the sensorimotor loop

The sensory-motor loop component consists of two controllers, for generating the grip force and lift force, and a plant model. *F*_*G*_ and *F*_*L*_ and given as inputs to the plant model which simulates the of the object and the fingers. The error between actual and desired object positions is fed back to the *F*_*L*_ controller. The *F*_*G*_ controller receives *F*_Gref_ as input and generates the *F*_*G*_(*T*) profile. *F*_Gref_ is generated by the second component, the BG component, by a DM process. The BG component is built on the lines of Actor-Critic models of the BG, where the utility function used instead of value function, and the dopamine signal, δ, is the temporal difference of utility [Equation (24)].

### The F_L_controller forms a loop with the plant

The controller gives *F*_*L*_ as input to the plant and receives position error as feedback. The controller is trained by GA as described in “The Precision Grip Control System”. The grip force controller is designed as an open-loop second order system that gives *F*_*G*_ as input to the plant (see The “Precision Grip Control System”). Plant dynamics is described in Appendix A. The controller and plant system, with its trained parameters, is then integrated with appropriately trained the BG models to simulate control and PD results (Ingvarsson et al., 1997; Fellows et al., 1998).

#### Simulation of Fellows et al. (1998) results

Incorporating the values of object mass (M_*o*_ = 0.33 kg) and friction (μ = 0.44) from Fellows et al. (1998), the controller and plant system trained above is used to calculate *V* and *h* functions (see “The Precision Grip Control System”). The *V* and *h* functions thus computed are explicitly modeled using RBFNNs (see “The Utility Function Formulation”). The *V* and *h* functions are combined to produce the utility function, which is used in the GEN method [see “Computing *U*(*F*_Gref_)”] to produce mean(*SGF*) and var(*SGF*) as outputs. The parameters of the GEN method must be calibrated to each experimental setup. Thus, the GEN parameters (*A*_*G/E/N*_, λ_*G/N*_and σ_*E*_) are trained by GA to produce the mean(*SGF*) and var(*SGF*) corresponding to the controls case. Then for the PD ON case, only δ_Lim_ and δ_Med_ are optimized (*A*_*G/E/N*_, λ_*G/N*_ and σ_*E*_ are unchanged). Simulated and experimental values of mean(*SGF*) and var(*SGF*) approximate the experimental mean(*SGF*) and var(*SGF*) (see Results: Figures 11, 12).

#### Simulation of Ingvarsson et al. (1997) results

The case of Ingvarsson et al. (1997) is a bit more complicated since it involves two friction conditions: silk and sandpaper. It also involves both PD ON and OFF unlike Fellows et al. (1998) which describes results for only PD ON. For the condition of sandpaper, object mass (M_*o*_ = 0.3 kg) and friction (μ = 0.94) are incorporated into the trained controller and plant system. *V* and *h* functions are computed and explicitly modeled using RBFNNs (“The Utility Function Formulation”) and Utility function is computed by combining *V* and *h*. The Utility function is used in the GEN method; the GEN parameters (*A*_*G/E/N*_, λ_*G/N*_ and σ_*E*_) are trained to minimize *CE*_GEN_[Equation (27), Figure 5] so as to calibrate the model for the sandpaper case of Ingvarsson et al. (1997). The trained GEN parameters are used for the PD OFF case and only δ_Lim_ is trained to optimize *CE*_GEN_. The same GEN parameters are again used for the PD ON case, where both δ_Lim_ and δ_Med_ are optimized. A similar procedure is followed for the “silk” case (M_*o*_ = 0.3 kg and μ = 0.44) of Ingvarsson et al. (1997) as outlined in Figure 13.

Risk-based decision making can arise in both motor and cognitive domains (Claassen et al., 2011). The present study deals with risk in motor domain. In this context, an interesting question naturally arises. Is there a correlation between risk-sensitivity in motor and cognitive domains. Does impaired risk-sensitivity in one domain carry over to the other? In other words, do PD patients show impaired decision making in motor and cognitive domains equally? In order to answer the above line of questioning, we propose to use a task, the Balloon Analog Risk Task (BART), which tests risk-sensitivity in cognitive domain (Claassen et al., 2011). We then propose to adapt that BART to the motor domain.

In BART, the subject is asked to press a key and inflate a virtual balloon displayed on the monitor. For every key press, the virtual balloon is inflated by a fixed amount and the subject earns a fixed number of points. The catch lies in that, on inflation beyond a threshold volume, the balloon bursts and the subject loses all the points. Knowing when to stop and redeem all the points earned so far involves risk based decision making.

The above task, which is a cognitive task, can be redesigned in terms of motor function, specifically in terms of PG performance. Just as in the BART task there is a threshold point at which the balloon bursts, in PG task there is a threshold grip force at which the object slips. In the redesigned BART task, the subject will earn more points as he/she grips the object with the grip force as close as possible to the slip force. Uncertainty can be incorporated by using objects that look identical but with different weights. It will be interesting to see possible parallels in performance of normal or PD patients, on both the cognitive and PG versions of the BART. If the above line of experimentation confirms that there is correlation between risk-sensitivity in motor and cognitive domains, it would place risk-based decision making approach to understanding PD on a stronger foundation.

## Author contributions

Ankur Gupta: Computational model development, analysis and manuscript preparation. Pragathi Priyadharsini B: Computational model development, analysis and manuscript preparation. V. Srinivasa Chakravarthy: Computational model development, analysis and manuscript preparation.

### Conflict of interest statement

The authors declare that the research was conducted in the absence of any commercial or financial relationships that could be construed as a potential conflict of interest.

## Appendix A

### PG model

#### Plant

The forces (*F*_*L*_ and *F*_*G*_) obtained from the two controllers are used for determining the kinetic (position, velocity and acceleration of finger and object). The plant model incorporates the *F*_*L*_and *F*_*G*_ for obtaining the net forces acting on both the finger (F_fin_) and object (F_*o*_), with the interaction based on with the interaction based on finger-object interface through friction (F_f_). The net force acting on finger and object is given in Equations (29, 30). Please note that *M*_fin_ is kept constant to M_*o*_/10 in the model.

(29) $$F_{\text{fin}}=F_{L}-F_{f}-M_{\text{fin}}g$$

(30) $$F_{o}=F_{f}+\;F_{n}-M_{o}g$$

When the object is resting on surface the net force on object is zero as there is no acceleration. So, the normal force is obtained by keeping F_*o*_ = 0 in Equation (30). When the object is lifted from the table the normal force becomes zero. F_*n*_ determination is given in Equation (31).

(31) $$F_{n}=\{\begin{array}{c} M_{o}g-F_{f},\text{ if }{\text{X}}_{o}=0\wedge M_{o}g>F_{f}\\ 0,\text{ else} \end{array}$$

The frictional force (F_*f*_) coupling the finger and object is given in Equation (32)

(32) $$F_{f}=\{\begin{array}{ll} \begin{array}{c} F_{\text{noslip}},\\ F_{\text{slip}}\;, \end{array} & \begin{array}{c} \text{if }F_{\text{noslip}}<F_{\text{slip}}\\ \text{ else} \end{array} \end{array}$$

Where, the *F*_slip_, representing the maximum frictional force that can be generated is given in Equation (33).

(33) $$F_{\text{slip}}=2\mu {\text{F}}_{G}$$

The F_*f*_ required to prevent slip is given in Equation (34)

(34) $$F_{\text{noslip}}=\frac{M_{o}M_{\text{fin}}}{M_{o}+M_{\text{fin}}}\left(\frac{F_{L}}{M_{\text{fin}}}-\frac{F_{n}}{M_{o}}\right)$$

According to Newton's second law of motion force is given as a product of mass and acceleration. So, Equations (29, 30) can also be represented as Equations (35, 36).

(35) $$F_{\text{fin}}=M_{\text{fin}}\frac{d^{2}X_{\text{fin}}}{dx^{2}}$$

(36) $$F_{o}=M_{o}\frac{d^{2}X_{o}}{dt^{2}}$$

The kinetic parameters can be obtained by integrating$\frac{d^{2}X_{o}}{dt^{2}}$ to obtain velocity and double integrated to obtain the position.

## Appendix B

### Training RBF

In order to determine *U* in PG performance we first need to identify the state and the reward signal. Since *F*_Gref_ is the key variable that decides the final outcome, *F*_Gref_ at trial (‘t’) is treated as a state variable.

As described earlier, calculation of *U*(*F*_Gref_(*t*)) requires *V*(*F*_Gref_(*t*)) and *h*(*F*_Gref_(*t*)) as explicit functions of *F*_Gref_(*t*). To this end we use data-modeling capabilities of neural networks to implement *V*(*F*_Gref_) and *h*(*F*_Gref_) as explicit functions of *F*_Gref_.

Using the values of *V*(*F*_Gref_) and *h*(*F*_Gref_) as output and *F*_Gref_ as input, an RBFNN (contains 60 neurons with the centroids distributed over a range [0.1 12] in steps of 0.2, and a standard deviation (σ_RBF_) of 0.7) was constructed and trained to approximate *V*(*F*_Gref_) and *h*(*F*_Gref_). For a given *F*_Gref_(*t*), a feature vector (Φ) is represented using RBFNN [Equation (37)].

(37) $$\phi_{m}(F_{\text{Gref}}(t))=\exp(-{\left(F_{\text{Gref}}(t)-\mu_{m}\right)}^{2}/\sigma_{m}^{2})$$

Here, for the m^th^ basis function, μ_*m*_ denotes the center and σ_*m*_ denotes the spread.

Using the ϕ that was obtained from Equation (37). The RBFNN weight for determining value, w_*V*_, is updated in Equation (38). Hence the value is the mean of all the *V*_*CE*_'s obtained on ${\hat{F}}_{\text{Gref}.}$

(38) $$\Delta w_{V}=\eta_{V}\;\Delta V_{CE}(F_{\text{Gref}})\;\phi (F_{\text{Gref}})$$

where η_*V*_ is the learning rate maintained to be 0.1, and the change in *V*_*CE*_ is given as in Equation (39).

(39) $$\Delta V_{CE}(F_{\text{Gref}})=e^{-CE({\hat{F}}_{\text{Gref}})}-e^{-CE(F_{\text{Gref}})}$$

The risk function *(h)* is then the variance in the Δ*V_CE_* as per Equation (40). Risk is the variance seen in all the *V*_*CE*_'s obtained on ${\hat{F}}_{\text{Gref}.}$

(40) $$\xi (F_{\text{Gref}})=\Delta V_{CE}{\left(F_{\text{Gref}}\right)}^{2}-h(F_{\text{Gref}})$$

The weights for risk function *w*_*h*_, is updated Equation (41)

(41) $$\Delta w_{h}=\eta_{h}\;\xi ({\text{F}}_{\text{Gref}})\;\phi (F_{\text{Gref}})$$

Here, η_*h*_ is the learning rate for risk function = 0.1 and ξ is the risk prediction error [Equation (40)].

From the trained RBFNN, *V*(*F*_Gref_) and *h*(*F*_Gref_) are calculated using Equations (42, 43), respectively.

(42) $$V(F_{\text{Gref}}\left(\text{t}\right))=w_{V}\phi \text{​}\left(F_{\text{Gref}}(t)\right)$$

(43) $$h(F_{\text{Gref}}\left(\text{t}\right))=w_{h}\phi \text{​}\left(F_{\text{Gref}}(t)\right)$$

## Abbreviations:

*A_G/E/N_*: Gains of Go/Explore/NoGo components of GEN equation in Equation (23)
BG: Basal Ganglia
CE: cost function to evaluate the performance of lift
*CE*_GEN_: Cost function for optimizing GEN parameters
*DA_hi_* and *DA_lo_*: thresholds at which dynamics switches between Go, NoGo and Explore regimes
DM: decision making
DP: Direct Pathway
*E*_*L*_: position error
*expt*: experiment
*F*_*f*_: frictional force
*F*_*G*_: grip force
*F*_Gref_: reference grip force
*F*_*L*_: Lift force
*F*_slip_: minimum force required to prevent the object from slipping
g: acceleration due to gravity
GEN: Go-Explore-NoGo
GPe: Globus Pallidus externa
GPi: Globus Pallidus interna
*h*: risk function
IP: Indirect Pathway
*K_P,L_, K_I,L_*, and *K_D,L_*: the proportional, integral and derivative gains of the Lift force controller
M: mass (subscript “O” and “fin” denote object and finger, respectively)
*M_p_*: maximum peak value of the response curve
PD OFF: Off medicated PD subjects
PD ON: On medicated PD subjects
PD: Parkinson's disease
PG: Precision Grip
PID: Proportional-Integral-Derivative controller
*r*: reward
RBFNN: Radial Basis Function Neural Networks
RL: Reinforcement Learning
*SGF*: Stable Grip Force
sim: simulation
SM: Safety margin
SNc: Substantia Nigra pars compacta
SNr: Substantia Nigra pars reticulata
STN: Subthalamic Nucleus
T: simulation time in milliseconds
*t*: trial
*T_p_*: peaking time of the response curve
*U*: Utility function
*V*: Value function
*X*/Ẋ/Ẍ: position/ velocity/acceleration (subscript “O” and “fin” denote object and finger respectively)
*X*: mean position (subscript “O” and “fin” denote object and finger respectively)
*X*_ref_: reference position
α: weight factor combining the value and the risk functions
δ: reward prediction error
δ_Lim_: clamped value of δ_*U*_
δ_Med_: added δ_*U*_ due to medication
δ_*U*_: change in Utility function
δ_*U*_: gradient of the Utility function
δ_*V*_: Temporal difference in value function
ζ: damping factor
λ_*G/N*_: sensitivities of the Go/NoGo component in Equation 23
μ: friction coefficient
μ_*m*_: means of RBFNN
ν: uniformly distributed noise
ξ: risk prediction error
π: policy
σ_*E*_: standard deviation used for the Explore component in Equation (23)
σ_*m*_: standard deviations for RBFNN
ϕ: feature vector
ψ: random variable uniformly distributed between -1 and 1
ω_*d*_: the damped natural frequency
ω_*n*_: natural frequency.
