# Supplementary material for: Computational model of precision grip in Parkinson's disease: a utility based approach
Source: Front Comput Neurosci. 2013 Dec 2;7:172. doi: 10.3389/fncom.2013.00172 (PMC3845796; doi:10.3389/fncom.2013.00172)
Supplement: Supplementary file 1 [file DataSheet1.PDF]

SUPPLEMENTARY MATERIAL A

The Genetic Algorithm (Goldberg et al., 1989) option set for optimization is given in the adjoining table. Optimization toolbox 6.0, MatlabR2011a, The Mathworks Inc. is used.

Option set for the GA tool.

**Option**	**Option set for determining F_L controller parameters**	**Option set for determining GEN parameters**
Population Size	100	20
Crossover fraction	0.8	0.8
Elite count	4	4
Generation time	1000	1000
Function tolerance	1 e-6	1 e-6
Cost function	CE	CE_{GEN}

CE= cost function to evaluate the performance of lift (eqn.(8)); CE_{GEN}= Cost function for optimizing GEN parameters (eqn.(27)); GEN=Go-Explore-NoGo (eqn.(23)); F_L: Lift force.

SUPPLEMENTARY MATERIAL B

Having only the value function for action selection:
In this section the only value function based approach is unable to generate an increased grip force for Parkinson's patients (25% and 50% clamping) when compared to controls (0% cell loss).

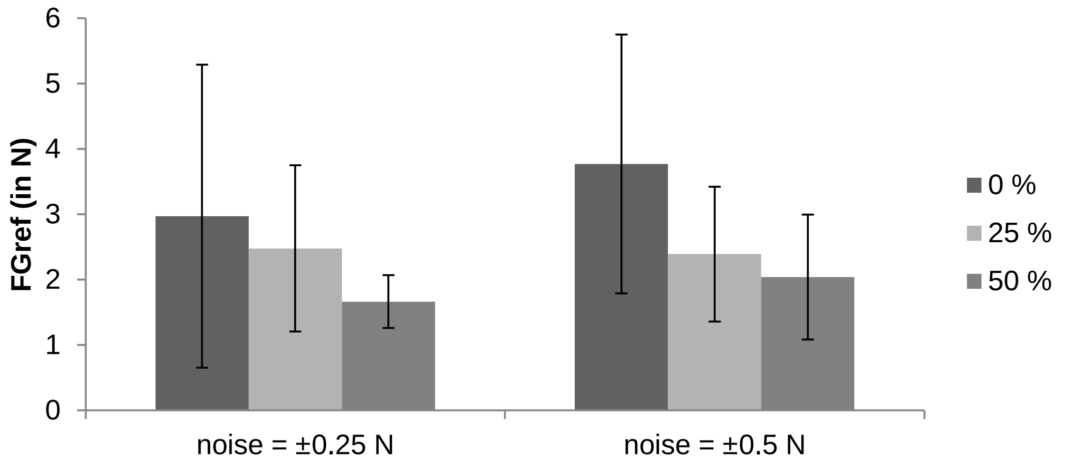

Comparison of GF generated under different cell loss conditions (0% resembling controls and 25% and 50% represent 25% and 50% clamping of δ in PD patients). The experimentally recorded values were 4.33N (for 0% clamping) and 7.72 N (for assumed loss of 25% or 50% clamping) Fellows et al (1998).In none of the cases (25% or 50% clamping) the required results could be reproduced.
